# Cocktail Cell‐Reprogrammed Hydrogel Microspheres Achieving Scarless Hair Follicle Regeneration

**DOI:** 10.1002/advs.202306305

**Published:** 2024-01-15

**Authors:** Shuaifei Ji, Yingying Li, Lei Xiang, Mingyue Liu, Mingchen Xiong, Wenguo Cui, Xiaobing Fu, Xiaoyan Sun

**Affiliations:** ^1^ Research Center for Tissue Repair and Regeneration affiliated to the Medical Innovation Research Department PLA General Hospital and PLA Medical College PLA Key Laboratory of Tissue Repair and Regenerative Medicine and Beijing Key Research Laboratory of Skin Injury Repair and Regeneration Research Unit of Trauma Care Tissue Repair and Regeneration Chinese Academy of Medical Sciences 2019RU051 Beijing 100048 P. R. China; ^2^ Department of Orthopaedics Shanghai Key Laboratory for Prevention and Treatment of Bone and Joint Diseases Shanghai Institute of Traumatology and Orthopaedics Ruijin Hospital Shanghai Jiao Tong University School of Medicine 197 Ruijin 2nd Road Shanghai 200025 P. R. China

**Keywords:** hair follicles, hydrogel, microspheres, regeneration, scarless

## Abstract

The scar repair inevitably causes damage of skin function and loss of skin appendages such as hair follicles (HF). It is of great challenge in wound repair that how to intervene in scar formation while simultaneously remodeling HF niche and inducing in situ HF regeneration. Here, chemical reprogramming techniques are used to identify a clinically chemical cocktail (Tideglusib and Tamibarotene) that can drive fibroblasts toward dermal papilla cell (DPC) fate. Considering the advantage of biomaterials in tissue repair and their regulation in cell behavior that may contributes to cellular reprogramming, the artificial HF seeding (AHFS) hydrogel microspheres, inspired by the natural processes of “seeding and harvest”, are constructed via using a combination of liposome nanoparticle drug delivery system, photoresponsive hydrogel shell, positively charged polyamide modification, microfluidic and photocrosslinking techniques. The identified chemical cocktail is as the core nucleus of AHFS. In vitro and in vivo studies show that AHFS can regulate fibroblast fate, induce fibroblast‐to‐DPC reprogramming by activating the PI3K/AKT pathway, finally promoting wound healing and in situ HF regeneration while inhibiting scar formation in a two‐pronged translational approach. In conclusion, AHFS provides a new and effective strategy for functional repair of skin wounds.

## Graphical abstract

Inspired by the natural process “seeding, germination, maturation, and harvest”, fibroblast fate‐regulating seed microspheres that can reset dermal fibroblast fate, inhibit scar signaling, activate DPC program and induce in situ hair follicle regeneration are developed by high‐throughput mechanism‐driven phenotype compound screening techniques, liposome nanoparticle drug delivery system, surface positive charge modification, microfluidic, and UV photopolymerization technology.

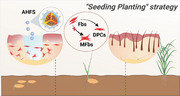

## 1 Introduction

Skin appendages, including hair follicles (HF), sweat glands, and sebaceous glands, are of great importance to skin function and homeostasis.^[^ ^1^ ^]^ However, scar repair after burns or a large skin defect often leads to the dysfunction of the native skin mesenchymal microenvironment, disruption to the overall skin architecture and function, as well as loss of skin appendages.^[^ ^2^ ^]^ Besides, skin scar‐caused HF loss also causes aesthetic concerns and further harm psychological health.^[^ ^3^ ^]^ It is estimated that 100 million people, owing to surgeries, get scars per year in high‐income nations alone, which poses an enormous burden on individuals and society.^[^ ^4^ ^]^ Commonly used hair regeneration techniques in clinic include hair transplantation as well as medical treatment (e.g., minoxidil).^[^ ^5^ ^]^ Such methods are often not satisfactory to patients, and the regenerated hair are also not durable.^[^ ^6^ ^]^ More importantly, these techniques are dependent on activating the vitality of innate HF to regenerate hair, so they cannot work in the scar repair where HF have been destroyed.^[^ ^7^ ^]^ Therefore, functional wound healing with in situ hair follicle regeneration and no scarring is a great concern in regenerative medicine. Currently, a panel of biomaterials that promote wound healing and hair follicle regeneration has been developed,^[^ ^8^ ^]^ such as microneedle targeting mechanical communication pathway^[^ ^9^ ^]^ and hydrogel with pulsatile inhibition of transforming growth factor β (TGFβ) signaling,^[^ ^10^ ^]^ etc. Nevertheless, these biomaterials primarily emphasize scar inhibition while disregarding the biological process of HF regeneration, which have little effect on the functional recapitulation of damaged skin.^[^ ^11^ ^]^ More importantly, these biomaterials face great obstacles in clinical applications. Therefore, it will be of great significance to develop a clinically available biomaterial that can, with a two‐pronged translational approach, both mobilize HF regeneration procedures while inhibit scar formation.

Understanding the biological processes involved in scarring formation and HF development may shed new light. During wound healing, the activation of dermal fibroblasts into myofibroblasts plays an essential role in scar formation.^[^ ^12^ ^]^ Myofibroblasts can produce and organize a massive collagen/extracellular matrix (ECM), resulting in dermal microenvironment disruption and skin appendage loss.^[^ ^13^ ^]^ Dermal papilla cells (DPC) are the dermal cell population located at the root of a hair follicle, which contributes to hair follicle development, growth, and regeneration, and they are developmentally originated from SRY‐box transcription factor 2 (SOX2)^+^ fibroblasts.^[^ ^14^ ^]^ Thus, regulation of fibroblast fate and activation of DPC programs may achieve scar‐free in situ HF regeneration. Chemical reprogramming techniques that reshape cell fate offer an attractive strategy for obtaining high‐quality fibroblast‐derived DPC,^[^ ^15^ ^]^ which encourages to find chemical cocktails that inhibit fibroblast signaling and activate DPC programming. Engineering biomaterials with micro/nanotechnologies or drug delivery have made a great process in situ reprogramming and tissue repair.^[^ ^16^ ^]^ Besides, biomaterial‐based regulation (e.g., gelatin and hyaluronic acid) may control cellular plasticity to accelerate reprogramming process.^[^ ^17^ ^]^ Hydrogel microspheres have the abundant functional groups, controllable physical properties, natural drug‐loading structures, and good biocompatibility.^[^ ^18^ ^]^ A recent study reported the injectable hyaluronic acid hydrogel microspheres‐loading PDGF and TGFβ3, could recruit endogenous stem cells and further induce in situ chondrogenic reprogramming for repairing osteoarthritis.^[^ ^19^ ^]^ Hydrogel microspheres have great potentials to be as drug carriers for promoting cellular reprogramming and inducing in situ tissue regeneration.^[^ ^20^ ^]^ Based on these advances, the construction of cocktail hydrogel microspheres inducing fibroblast‐to‐DPC reprogramming is expected to achieve in situ HF regeneration with reduced scarring.

**Figure 1:**
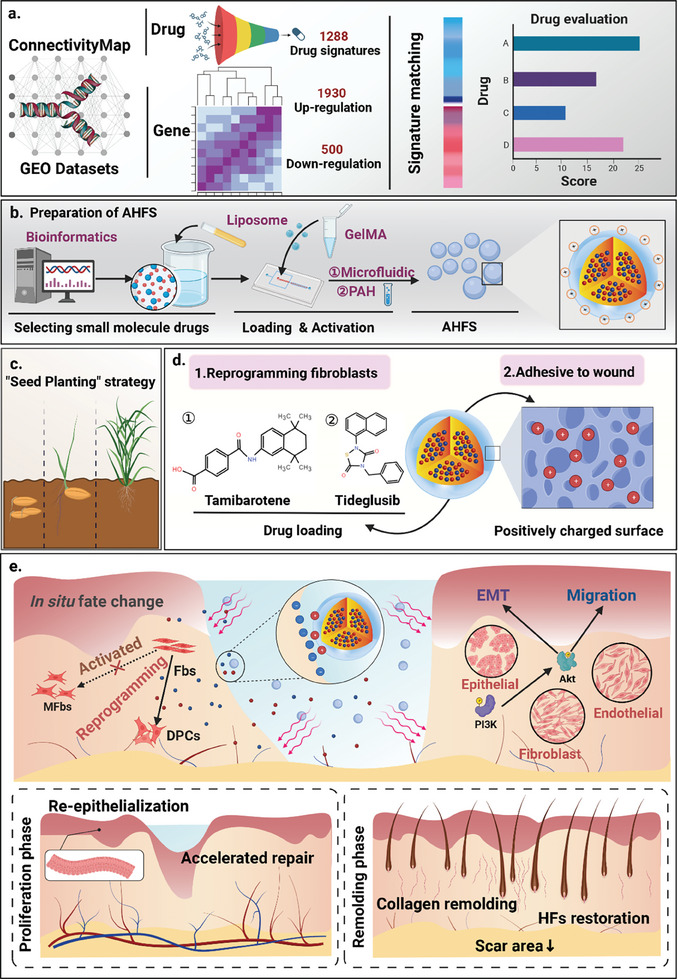
Schematic illustration. a) The principle of high‐throughput mechanism‐driven phenotype compound screening; b) Preparation of AHFS for in situ reprogramming; c) “Seed Planting” strategy for in situ hair follicle regeneration.; d) The characteristics of AHFS; e) AHFS promote wound healing, re‐epithelialization, in vivo fibroblast‐DPC transition, scar‐free repair and in situ hair follicle regeneration. Fbs, fibroblasts; MFbs, myofibroblasts; EMT, epithelial to mesenchymal transition.

Here, the clinically available drug tideglusib (Ti) and tamibarotene (T) were identified as the potential chemicals inhibiting fibroblast signaling and activating DPC fate by high‐throughput mechanism‐driven phenotype compound screening (**Figure** **1**a). Then, inspired by the natural process that “seeding, germination, maturation and harvesting”, artificial hair follicle seeding (AHFS) hydrogel microspheres with the ability to induce in situ fibroblast‐to‐DPC conversion were developed (Figure 1b,c). In this study, we used a liposome nanodrug delivery system to form the cocktail nucleus of AHFS to drive the transition of fibroblasts to DPC and enhance the ability of wound repair cells. The extracellular matrix component as the shell of AHFS provides a 3D environment suitable for tissue compatibility and cellular plasticity regulation.^[^ ^17c^ ^]^ Considering the characteristics of the electric field of the wound, we modified AHFS with polyamide to make its surface become a positive charged environment, giving AHFS the ability to adhere to the skin wound, and finally achieving the purpose of non‐invasive treatment (Figure 1d). Finally, size‐uniformed and dispersed seed microspheres were prepared based on microfluidic technology and UV crosslinking technology. AHFS are the cell reprogramming hydrogel microspheres that can adhere to wound site. AHFS works to regulate fibroblast fate, inhibit scar formation signaling, and simultaneously activate DPC programming as well as further induce in situ HF regeneration, through slow release of the chemical cocktail (Ti+T). Besides, seed microspheres AHFS can also enhance the ability of endogenous wound‐resident cells, such as epidermal cell and vascular endothelial cells, greatly promoting wound healing (Figure 1e). Collectively, AHFS, the seed hydrogel microspheres capable of “germinating” to produce HF, may provide a novel and effective platform for functional skin repair without scars.

## 2 Results and Discussion

### 2.1 Generation of AHFS with Long‐Acting DPC‐Fate Inducing Capacity

**Figure 2:**
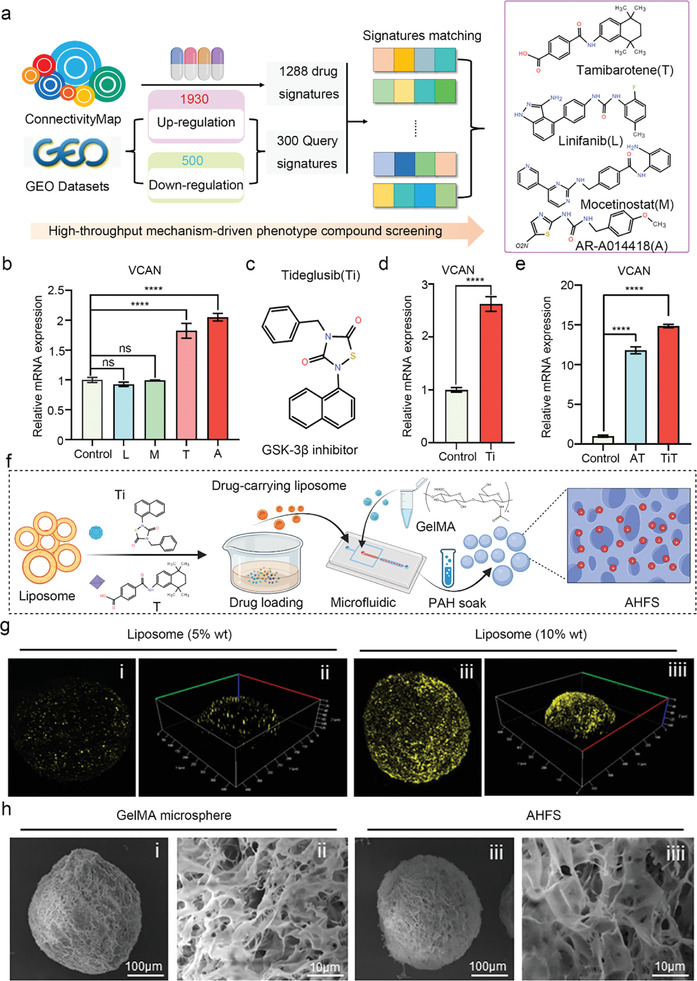
The preparation of AHFS with the ability to reset fibroblast fate. a) Transcriptomic analysis between HDF and primary DPC, high‐throughput mechanism‐driven phenotype compound screening, and the predicted drug candidates for fibroblast‐DPC reprogramming. b) HDF were treated with the predicted drug candidates for 5 days. qRT‐PCR analysis of transcriptional expression of the DPC‐associated genes *VCAN* after the drug treatment. L (Linifanib, 1 µmol L^−1^), M (Mocetinostat, 1 µmol L^−1^), T (Tamibarotene, 5 µmol L^−1^), A (AR‐A014418, 10 µmol L^−1^). c) The information of Tideglusib (Ti) structure. d) HDF were treated with Ti (5 µmol L^−1^) for 5 days. mRNA level of *VCAN* after Ti treatment. e) HDF were treated with A+T or Ti+T for 5 days. qRT‐PCR analysis of transcriptional expression of *VCAN* after these treatments, showing the optimal drug combination for activating DPC fate. All data in qRT‐PCR analysis are expressed as mean ± S.D; n = 3. ns, not significant, ^**^ *p* < 0.01, ^****^ *p* < 0.0001. f) Illustration of the fabrication of adhesive microspheres for in situ reprogramming. Ti and T were loaded in liposome, and photopolymerizing GelMA and the liposome carrying Ti and T by microfluidic technology to fabricate adhesive microspheres that can induce the conversion of fibroblast to DPC. g) LCSM images of liposome in 5% wt (i and ii) and 10% wt (iii and iiii). h) SEM images of GelMA microspheres (i: overall view, ii: local view) and AHFS microspheres (iii: overall view, iiii: local view).

The activation of dermal fibroblasts into myofibroblasts in response to wound signals during wound repair plays an essential role in skin scar formation.^[^ ^21^ ^]^ DPC, originating from SOX2^+^ fibroblasts, and contributes to HF development, growth, and regeneration.^[^ ^14^ ^]^ Considering the similar cell origin, theoretically, it is very feasible to convert human dermal fibroblasts (HDF) to DPC by chemical‐induced cellular reprogramming. To identify the chemical cocktail nucleus of AHFS that can convertHDF to DPC, the high‐throughput mechanism‐driven phenotype compound screening technology, which aims to discovery of potential drugs that may promote or inhibit the biological process by matching the differentially expressed gene (DEG) of target cells and desired cells with cMap database, was used.^[^ ^22^ ^]^ Transcriptomic analysis between HDF and primary DPC from the GEO database showed satisfactory uniformity and clustering in these two groups (Figure S1a–c, Supporting Information). The DGE profiling revealed there were 1930 up‐regulated genes and 500 down‐regulated genes in the comparison of primary DPC and HDF (Figure S1d, Supporting Information), and the up‐regulated genes were mainly enriched in the cytoskeleton and cell migration (Figure S1e, Supporting Information), stem cell function and osteogenic ability (Figure S1f, Supporting Information), hair growth and skin development (Figure S1g, Supporting Information), as well as interleukin and chemokines secretion (Figure S1h, Supporting Information). Through matching the DGE and 1288 drug signatures of cMap, a total of 13 drugs with the cMap score >90 were initially identified (**Figure** **2**a; Figure.S2a, Supporting Information), and the details of these drugs are summarized in Table S1 (Supporting Information). Comprehensively, considering the cMap scoring and targeting mechanisms, 4 drugs without skin sensitization that is likely to convert HDF to DPC, including Linifanib (Platelet derived growth factor receptor inhibitor, L), AR‐A014418(Glycogen synthase kinase 3 β inhibitor, A), Mocetinostat (Histone deacetylase inhibitor, M) and Tamibarotene (Retinoid receptor agonist, T), were selected in the second screening (Figure 2a; Figure S2b, Supporting Information). After treatment for 5 days, gene expression profiling analysis by qRT‐PCR revealed that A and T can increase the mRNA level of specific DPC marker *Versican* (*VCAN)*, while L and M not (Figure 2b), suggesting A and T may drive fibroblast fate toward DPC. The previous studies also reported that chemicals that inhibit glycogen synthase kinase 3 β signaling^[^ ^23^ ^]^ and activate retinoic acid metabolic pathway^[^ ^24^ ^]^ contributed to driving DPC program, which indirectly supported the role of A and T in DPC property activation. Besides, chemical screening has relied on literature reports and experimental trials to identify useful drug combinations, resulting in a lot of efforts and poor results.^[^ ^25^ ^]^ This work demonstrated the feasibility of high‐throughput mechanism‐driven phenotype compound screening technology in chemical reprogramming, which accelerated the drug discovery in fibroblast‐to‐DPC conversion. Then, considering the fact that T is FDA‐approval drug while A not, in order to increase the clinical translational potential, Tideglusib (Ti), another FDA‐approved GSK3β inhibitor that has been reported to facilitate wound healing,^[^ ^26^ ^]^ was selected to replace A (Figure 2c). The qRT‐PCR showed Ti can also activate the *VCAN* gene (Figure 2d), and meanwhile, the *VCAN* mRNA level in the combination of Ti+T(TiT) was further increased, and higher than that of A+T (AT) treatment (Figure 2e). Therefore, these two FDA‐approval drugs, Ti and T, consist the chemical cocktail nucleus of AHFS to induce HDF‐to‐DPC transition.

Considering the role of biomaterials in cell plasticity and its application in tissue repair,^[^ ^17b^ ^]^ seeking good biomaterial carrier loading TiT is essential for fabricating AHFS. It has been widely reported that liposome is an ideal platform for fat‐soluble drug loading and slow release.^[^ ^27^ ^]^ Hyaluronic acid and gelatin, as the major component of ECM, have proven effective in regulating key cellular processes and behaviors, including proliferation, differentiation, and the inflammatory response,^[^ ^28^ ^]^ which may contribute to cellular reprogramming.^[^ ^29^ ^]^ Adhesive seed microsphere AFHS with TiT cocktail nucleus and positively charged surface modification were developed through liposome nanodrug carrier system, microfluidic technology, photoresponsive crosslinking technology, and poly allylamine hydrochloride (PAH) activation (Figure 2f). The liposomes were prepared by the modified film dispersion method, and the monodisperse liposomes were obtained after repeated ultrasonic treatment during the rehydration process. The laser scanning confocal microscope (LSCM) based on 3D reconstructed images showed 10% wt liposomes perform well than 5% wt ones (Figure 2g). The encapsulation of liposomes can protect fat‐soluble drugs and extend the half‐life of drugs,^[^ ^30^ ^]^ thus 10% wt liposomes as TiT carriers to form core nucleus (liposome@TiT) of the seed microsphere AFHS. Field emission scanning electron microscopy (SEM) showed that AHFS possessed an intact porous structure similar to GelMA microspheres, which was of great significance to drug release (Figure 2h). Comprehensively, it is reasonable that AHFS can slowly release TiT to in vitro reset fibroblast fate, in vivo inhibit scar formation, and at the same time, promote in situ HF regeneration. Therefore, there is a need to implement performance characterization and function verification of AHFS.

### 2.2 Physicochemical Properties of AHFS

**Figure 3:**
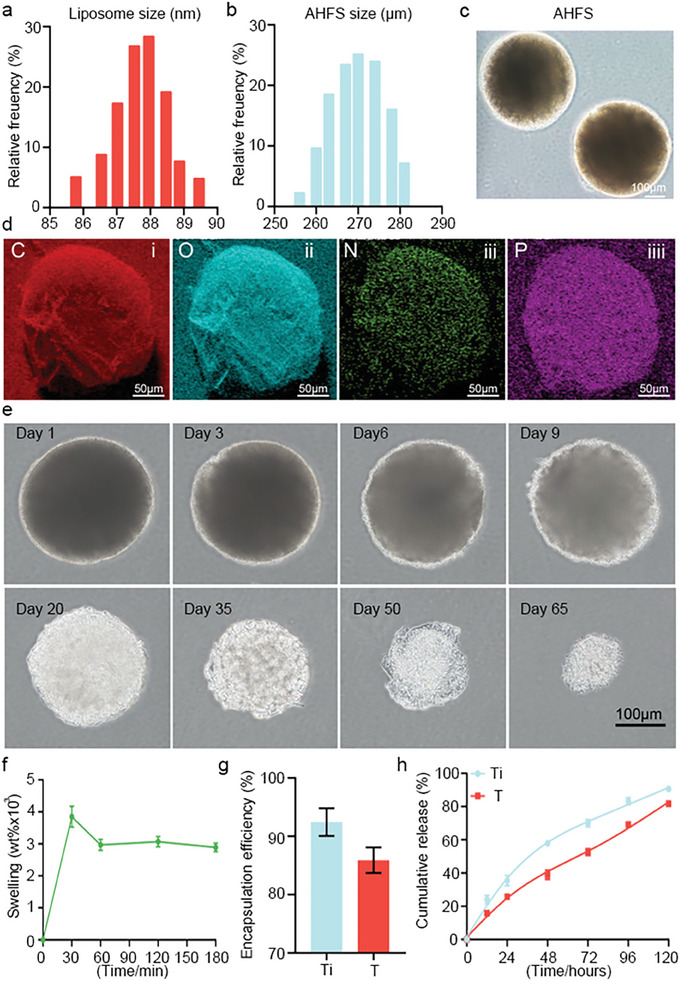
Physicochemical properties of AHFS microspheres. a) Particle size distribution of Liposome carrying TiT. b) Particle size distribution of AHFS microspheres. c) Bright field images of AHFS microspheres. d) Elemental mapping images of AHFS microspheres. e) Morphological changes of the AHFS microspheres during degradation tests. f) The swelling ratio of AHFS microspheres. g) The encapsulation efficiency of Ti and T in AHFS microspheres. h) Release curves of Ti and T releasing from AHFS microspheres.

Appropriate microsphere diameter was important for a 3D scaffold, and in general, 200–300 µm microsphere diameters were recommended.^[^ ^31^ ^]^ To get well‐sized AHFS seed microspheres, the core nucleus of AFHS (liposome@TiT) size diameter was 87.7±1.23 nm (**Figure** **3**a). The micromorphology of liposome@TiT was shown in Figure S3a (Supporting Information). The diameter of the monodisperse AHFS microsphere was 268.62±8.8 µm (Figure 3b). The light microscopy showed AHFS prepared by microfluidic technology was uniform in size and shape (Figure 3c). The elemental mapping based on LSCM 3D reconstructed image demonstrated that AHFS was enriched by uniformly distributed carbon (C), oxygen (O), nitrogen (N), and phosphorus (P) (Figure 3d). Based on the electric field environment of skin wounds,^[^ ^32^ ^]^ the AHFS with positively charged polyamide modification was designed to adhere to skin wounds electrostatically and achieve non‐invasive treatment. The results exhibited AHFS can combined with negatively charged microsphere via electrostatic effect (Figure S3b, Supporting Information), and the zeta potential test of the negatively charged microsphere and AHFS was shown in Figure S3c (Supporting Information). Besides, MS (microspheres) without wound adhesion were easily washed off by water, while AHFS seed microspheres can adhere better (Figure S3d, Supporting Information). Quantitative analysis suggested more AHFS seed microspheres remained on the skin wound even after washing (Figure S3e, Supporting Information). These results demonstrated that AHFS seed microspheres are positively charged and can adapt well to skin wounds, which was of great significance to wound repair independent of invasive injection. For a drug‐releasing 3D scaffold, another important characteristic is appropriate biodegradability.^[^ ^33^ ^]^ The light microscopy suggested that AHFS seed microspheres underwent a gradual degradation process, and the support structure of AHFS microspheres was still complete on day 20, and almost dissolved by day 50 (Figure 3e). The degradation rate of AHFS seed microspheres can cover the time well of fibroblasts‐myofibroblast transition and the cycle of wound repair with hair follicle regeneration. Owing to the sponge‐like 3D network structure consisting of hydrophilic polymer groups, the microspheres have the ability to imbibe a lot of fluids.^[^ ^34^ ^]^ When incubated in deionized water for 30 min, AHFS seed microspheres swelled to 3846.3±328.1wt%, then decreased to 2970±171.1wt% in 1 h, and finally reached stabilization (2889±134.1wt%) (Figure 3f). Because swelling promotes TiT to be absorbed into the microsphere network, the swelling capacity contributed to drug uptake. The swelling process was relatively gentle, which have little effect on TiT stability. Drug loading evaluation showed that the loading efficiency of Ti and T in microspheres was 92.8±2.9% and 85.9±2.2%, respectively (Figure 3g). Drug release analysis revealed Ti and T exhibited continuous release trends, and encapsulated drugs released 80%−95% after 5 days (Figure 3h). The above results suggested that chemical‐induced *VCAN* gene activation in fibroblasts required 5 days, so the drug release time may be favorable. Thus, AHFS may have the potential to disturb fibroblasts‐myofibroblast transition and drive DPC reprogramming.

### 2.3 Biocompatibility of AHFS with Wound‐Resident Cells

**Figure 4:**
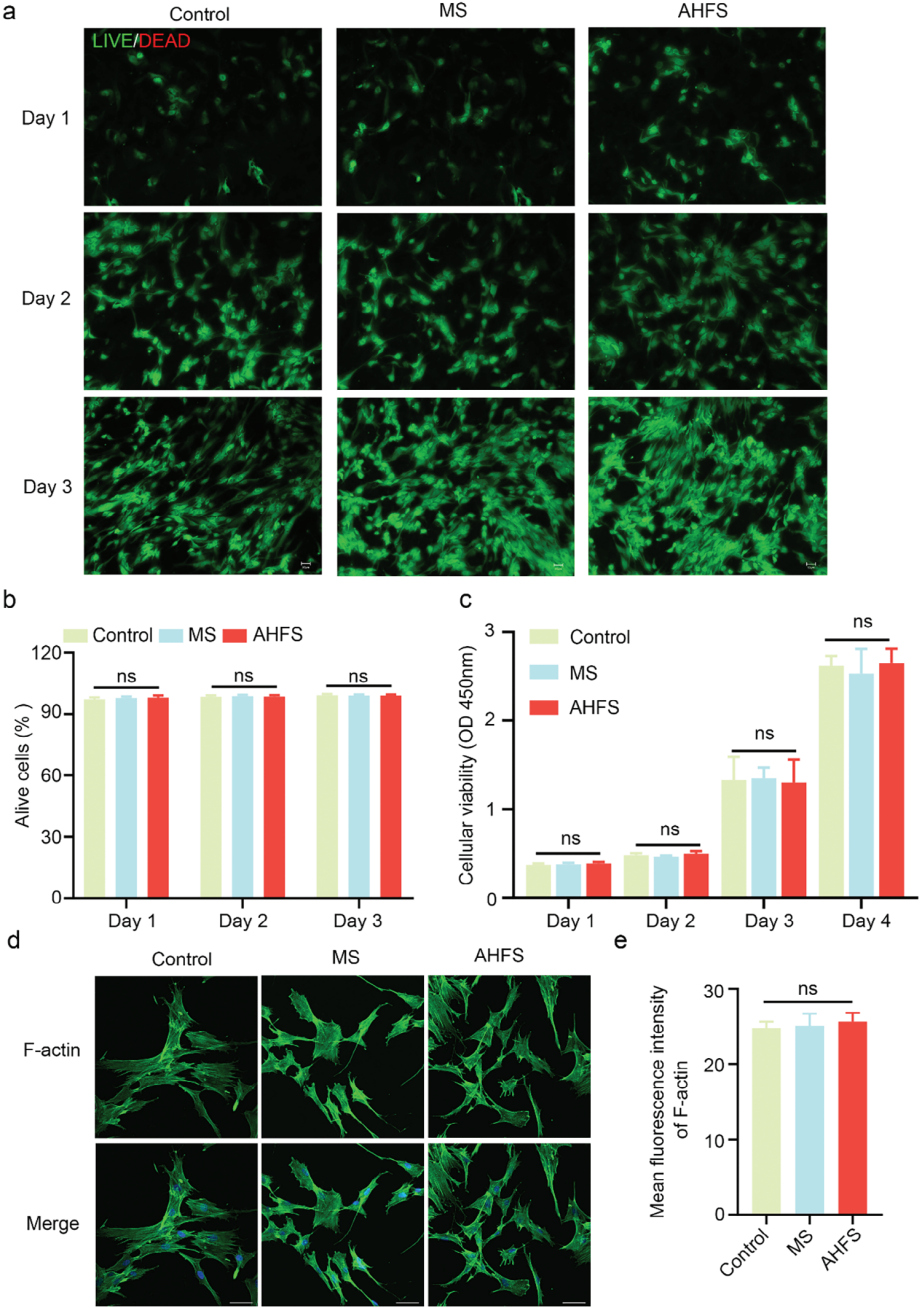
In vitro biocompatibility of AHFS. a) Images of live/dead cell staining on day 1, 2, and 3, among control, MS microsphere and AHFS microsphere group. Scale bar = 50 µm. b) Quantitative analysis for the percentage of alive cells in control, MS microsphere, and AHFS microsphere group. ns, not significant. c) CCK‐8 assay of control, MS microsphere, and AHFS microsphere group on day 1, 2, 3, and 4. d) Cytoskeleton staining after incubating with MS microsphere and AHFS microsphere. Scale bar = 50 µm. e) Quantitative analysis of F‐actin fluorescence intensity in control, MS microsphere, and AHFS microsphere group. ns, not significant.

Good biocompatibility is a prerequisite for the clinical applications of biomaterials.^[^ ^35^ ^]^ As a 3D scaffold for skin wound treatment, AHFS microspheres should be well‐biocompatible with the targeted cells. Live/dead staining assays showed that fibroblasts in all three groups were alive on days 1, 2, and 3 after co‐culture (**Figure** **4**a). The results of live/dead cell quantitative analysis suggested no statistical difference was observed in the percentage of alive cells between the groups (Figure 4b). Cell Counting Kit 8 (CCK8) test also showed consistent results, absorbance values were no statistical difference between the three groups after 1, 2, 3, and 4 days of co‐culture with AHFS (Figure 4c). Cytoskeleton analysis by immunofluorescence staining suggested both MS and AHFS didn't damage the fibroblast cytoskeleton (Figure 4d), and the quantitative analysis results also showed there was no statistical difference in F‐actin mean fluorescence intensity between all the groups (Figure 4e). Of note, with increasing cultivation time, AHFS may induce cellular reprogramming, and alter the fibroblast morphology and cytoskeleton toward DPC. As other wound‐resident cells, epidermal keratinocytes and vascular endothelial cells also play essential roles in wound healing.^[^ ^36^ ^]^ Thus, the low cytotoxicity of AHFS to these cells also needs to be evaluated, and human epidermal keratinocytes (HEK) and human umbilical vein endothelial cells (HUVEC) were used as reliable cell models. Live/dead staining assays suggested that the vast majority of HEK remained viable on days 1, 2, and 3 after AHFS treatment, and there was no statistical difference between the groups (Figure S4a,b, Supporting Information). Similar results were also observed in the co‐culture system of AHFS microspheres and HUVEC (Figure S4c,d, Supporting Information). Collectively, these results revealed that AHFS seed microspheres had favorable biocompatibility with wound‐resident cells, and good safety for future clinical applications.

### 2.4 AHFS Induce Fibroblast‐To‐DPC Conversion In Vitro

**Figure 5:**
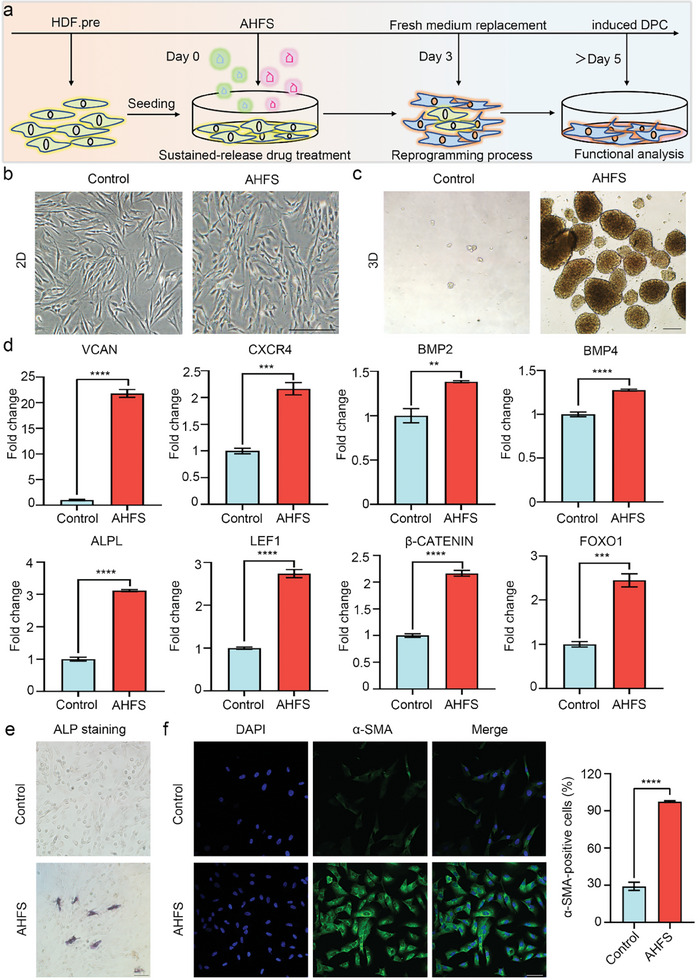
AHFS microsphere for in vitro reprogramming of fibroblast into DPC. a) Scheme of AHFS microsphere‐based reprogramming procedure. HDF were treated with AHFS microsphere for indicated days. b) Phase contrast images showing the morphological characteristics of HDF and AHFS microsphere‐induced DPC in 2D culture. Scale bar = 200 µm. c) Multicellular sphere formation assay of HDF and AHFS microsphere‐induced DPC in 3D culture. Scale bar = 100 µm. d) qRT‐PCR analysis of transcriptional expression of DPC‐associated maker *VCAN, CXCR4, BMP2, BMP4, ALPL, LEF1, β‐CATENIN* and *FOXO1* in HDF and AHFS microsphere‐induced DPC after 5 days of induction. All data in qRT‐PCR analysis are expressed as mean ± S.D; n = 3. ns, not significant, ^**^ *p* < 0.01, ^***^ *p* < 0.001, ^****^ *p* < 0.0001. e) ALP staining showing the ALP‐positive response in HDF and AHFS microsphere‐induced DPC. Scale bar = 100 µm. f) Representative immunofluorescence and quantitative analysis of DPC marker 𝛼‐SMA in HDF, and AHFS microsphere‐induced DPC at day 5 after AHFS microsphere treatment. ^****^ *p* < 0.0001. Scale bar = 50 µm.

To test the function of AHFS microspheres, we designed the strategy to systemically reprogram HDF to DPC by AHFS microsphere treatment (**Figure** **5**a). After induction of 5 days, in contrast to the elongated morphology in HDF, the AHFS‐treated cells exhibited smaller morphology, while MS without TiT microspheres didn't change HDF morphology (Figure 5b; Figure S5a, Supporting Information). The ability to form multicellular spheres has been reported as the characteristic of DPC.^[^ ^37^ ^]^ Multicellular sphere formation assay showed that, under the low‐adhesive culture conditions, the AHFS‐treated cells were able to form 3D multicellular spheres, while HDF and MS‐treated cells did not (Figure 5c; Figure S5b, Supporting Information). Of note, the gene expression profiling by qRT‐PCR revealed the mRNA level of VCAN in AHFS‐treated cells was further increased and higher than that in TiT‐treated cells (Figures 5d and 2e). Other DPC‐associated markers, including cell migration‐related functional markers (e.g., *CXCR4*), osteogenesis markers (e.g., *BMP2*, *BMP4*, and *ALPL*) and hair growth‐associated transcription factors (e.g., *LEF1*, *β‐CATENIN*, and *FOXO1*) also exhibited elevated mRNA levels after AHFS treatment (Figure 5d). Likewise, MS without TiT treatment did not activate DPC markers (Figure S5c, Supporting Information). These results showed MS did not induce fibroblast‐to‐DPC conversion, but promote chemical reprogramming, which may be attributable to its effect on cell plasticity.^[^ ^38^ ^]^ The alkaline phosphatase (ALP) activity is another DPC‐specific feature that distinguishes dermal fibroblasts.^[^ ^39^ ^]^ ALP staining was used to evaluate the ALP activity of AHFS‐treated cells, and the results suggested that, compared to control, AHFS can activate ALP positive response (Figure 5e). The immunostaining further demonstrated percentage of AHFS‐treated cells expressing α‐SMA was 97.48±0.71%, significantly higher than that of HDF (29.10±3.21%) (Figure 5f). These results demonstrated that AHFS‐treated cells have exhibited phenotypic characteristics of DPC.

**Figure 6:**
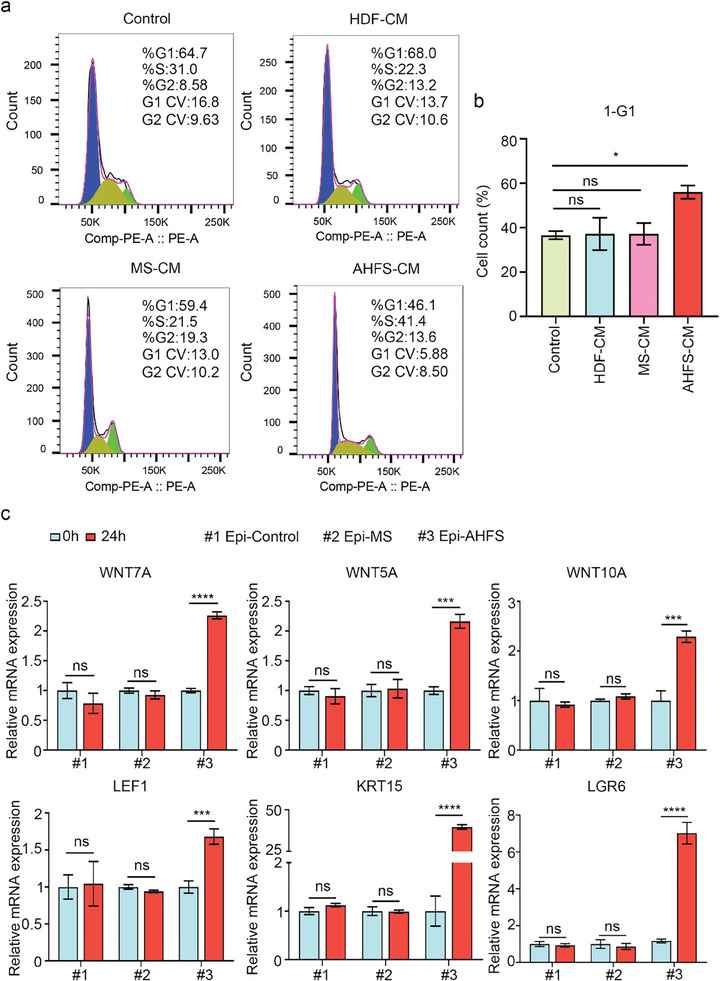
Functional characteristics of AHFS‐induced DPC. a) Cell cycle analysis of the effect of AHFS microsphere‐induced DPC on HEK. G1 phase reflects the proliferation arrest, and the high percentage of 1‐G1 cells means active proliferative activity. Control, HEK without treatment; HDF‐CM group, HEK with the treatment with condition medium from HDF; MS‐CM group, HEK with the treatment with condition medium from MS treated‐HDF; AHFS‐CM group, HEK with the treatment with condition medium from AHFS‐induced DPC. b) Quantitative analysis of the percentage of 1‐G1 cells in control, HDF‐CM group, MS‐CM group, and AHFS‐CM group. ns, not significant, ^*^ *p* < 0.05. c) qRT‐PCR analysis of transcriptional expression of hair follicle‐associated genes in the dermal‐epidermal aggregation after 24 h. Epi‐Control, the aggregation of HEK and HDF; Epi‐MS, the aggregation of HEK and MS‐treated HDF; Epi‐AHFS, the aggregation of HEK and AHFS‐induced DPC. not significant, ^***^ *p* < 0.001, ^****^ *p* < 0.0001.

Then, there was also a need to evaluate whether AHFS‐treated cells possessed functional characteristics like DPC. The unique function of DPC was that DPC can communicate with epidermal keratinocytes, which was essential for hair morphogenesis and cycling.^[^ ^40^ ^]^ In epithelial‐mesenchymal interactions, DPC can promote epidermal keratinocyte proliferation under co‐culture conditions.^[^ ^41^ ^]^ Therefore, an indirect co‐culture system was established to test whether AHFS‐treated cells can contribute to HEK proliferation. The cell cycle analysis showed that, compared with the HEK control, the conditioned medium (CM) from HDF and MS‐treated HDF did not affect the percentages of HEK in the G1 phase, while the CM from AHFS‐treated cells can significantly decrease the percentages of HEK in the G1 phase, with higher 1‐G1, indicating AHFS‐treated cells can facilitate epidermal keratinocyte mitosis and proliferation (**Figure** **6**a,b). Then, pre‐aggregation of DPC and epidermal keratinocyte can activate the hair regeneration‐related gene (e.g., *KRT15, WNT7A, WNT10A, WNT5A LEF1*, and *LGR6*).^[^ ^42^ ^]^ Using ultra‐low attachment culture to form dermal‐epidermal aggregation, and qRT‐PCR results showed that the increased^[^ ^21^ ^]^ mRNA levels of hair regeneration‐related genes were only observed in AHFS‐treated cell‐HEK aggregation, and these genes were not activated among the remaining groups (Figure 6c). These results suggested that AHFS‐treated cells possessed functional characteristics of DPC. Therefore, AHFS can drive the changes in HDF fate and induce fibroblasts‐DPC transition.

### 2.5 AHFS Induce Fibroblast‐To‐DPC Conversion by Activating PI3K/AKT Signaling Pathway

**Figure 7:**
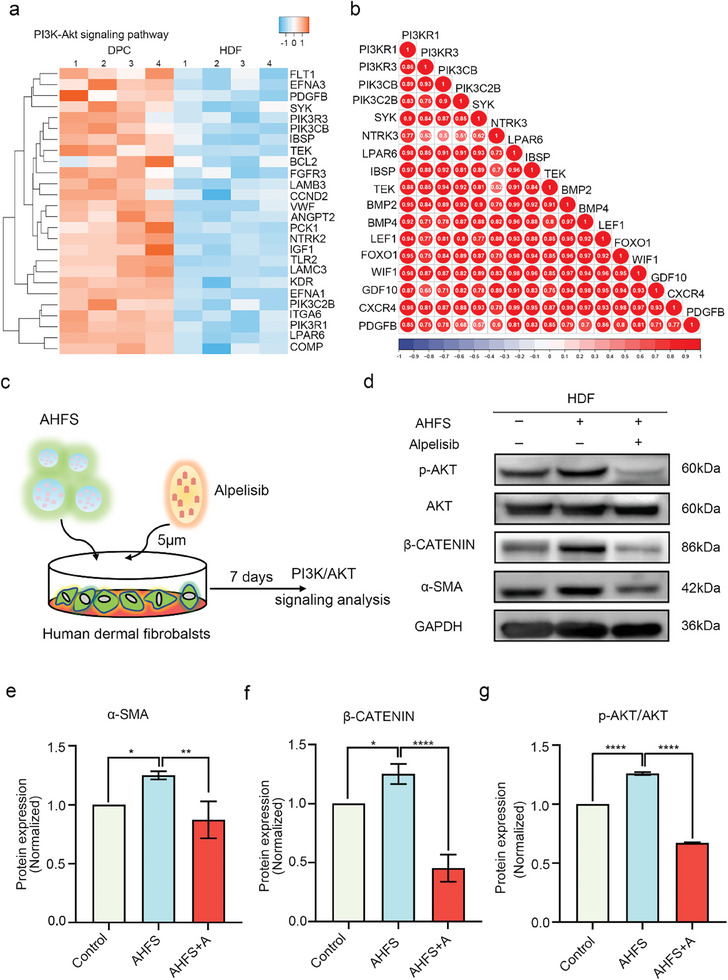
AHFS‐induced fibroblast‐DPC transition is mediated by PI3K/AKT signaling pathway. a) Heatmap analysis of PI3K/AKT signaling pathway‐associated genes between primary DPC and HDF. b) Correlation heatmap showing the positive relation of PI3K/AKT signaling pathway to DPC genes. c) PI3K inhibitor alpelisib was used to test the role of PI3K/AKT signaling pathway in AHFS‐induced fibroblast‐DPC transition. d) Western blot analysis of phosphorylation AKT, AKT and DPC marker α‐SMA and β‐CATENIN in HDF with or without AHFS and AHFS+ alpelisib treatment. e, f and g) Qualification of α‐SMA, β‐CATENIN, and p‐AKT/AKT immunoblots was performed using Image J software. GAPDH was used as internal loading control. ^*^ *p* < 0.05, ^**^ *p* < 0.01, ^****^ *p* < 0.0001.

Previous research has reported that PI3K/AKT signaling pathway was crucial for the characteristics and function of DPC.^[^ ^43^ ^]^ Activation of PI3K/AKT signaling pathway can increase growth properties in human DPC spheroids,^[^ ^44^ ^]^ improve trichogenous gene expression of DPCs and maintain and restore hair inductivity of DPCs.^[^ ^43^ ^]^ PI3K/AKT signaling pathway has served as a targeted mechanism for multiple interventions to promote hair follicle regeneration.^[^ ^45^ ^]^ The KEGG analysis suggested the DEG between primary DPC and HDF was significantly enriched in PI3K/AKT signaling pathway (Figure S6a, Supporting Information). Heat map analysis showed that compared to HDF, the genes of the PI3K/AKT signaling pathway were highly expressed in DPC (**Figure** **7**a). Spearman correlation coefficient matrix revealed that PI3K/AKT signaling pathway was positively associated with DPC‐related markers (e.g., *BMP2, BMP4, LEF1, FOXO1, WIF1, GDF10, CXCR4*, and *PDGFB*) (Figure 7b). These results indicated that, in contrast to HDF, there was activated PI3K/AKT signaling pathway in DPC, and this pathway may contribute to DPC characteristics and properties. Based on that, it was hypothesized that PI3K/AKT signaling pathway may get involved in AHFS‐mediated fibroblasts‐DPC conversion, and thus the inhibitor of PI3K/AKT signaling pathway (Alpelisib) was used to verify the hypothesis (Figure 7c). The microscope results showed that the cells in the AHFS+Alpelisib group did not exhibit smaller morphology but elongated morphology similar to HDF (Figure S6b, Supporting Information). Multicellular sphere formation assay suggested that the ability to form the multicellular sphere of cells was reduced in the AHFS+Alpelisib group, which revealed inhibiting PI3K/AKT signaling pathway can decrease AHFS‐treated cell function (Figure S6c, Supporting Information). The western blot results showed that AHFS alone increased AKT phosphorylation level, which meant activated PI3K/AKT signaling pathway, and enhanced the protein level of DPC‐specific markers (e.g., *β‐CATENIN* and *α‐SMA*) (Figure 7d–g). However, in the AHFS+ Alpelisib group, AKT phosphorylation level and β‐CATENIN and α‐SMA protein levels were significantly decreased, indicating PI3K/AKT signaling pathway and DPC characteristics were inhibited (Figure 7d–g). These results demonstrated that PI3K/AKT signaling pathway was indeed essential for the acquisition of DPC characteristics, and participated in the AHFS‐induced reprogramming of HDF into DPC.

### 2.6 AHFS Enhance Wound‐Resident Cell Ability and Facilitate Rapid Wound Healing

**Figure 8:**
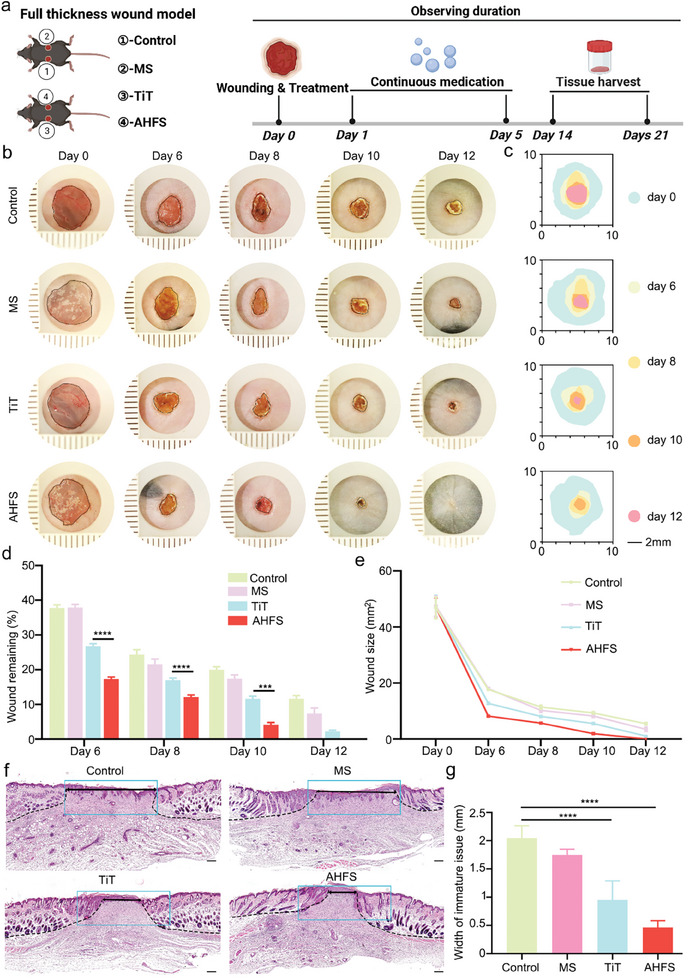
AHFS accelerate wound healing. a) Schematic illustration representing the experimental procedure of wound healing study. b) Representative macroscopic illustration of wound healing at indicated days in control, MS, TiT and AHFS‐treated mices. c) Traces of wound‐bed closure during 12 days for four groups. d) Wound remaining rate in control, MS, TiT and AHFS‐treated mice on day 6, 8, 10, and 12. n = 5 per group. e) Wound size over time in control, MS, TiT and AHFS‐treated mice. n = 5 per group. f) Hematoxylin and eosin (H&E) stained sections of wounds in four groups at day 14 showing width of immature tissue and hair follicle regeneration. Scale bar = 200 µm. g) Qualification of width of immature tissue in four groups at day 14. n = 5 per group. ^****^ *p* < 0.0001.

Because AHFS was a biomaterial for skin wound, its effect on other wound‐resident cells (e.g., epidermal cells and vascular endothelial cell) also need to be tested. After the incubation of AHFS and HEK for 48 h, the gene expression profiles by qRT‐PCR revealed that the epithelial marker E‐cadherin was significantly reduced in AHFS‐treated HEK, while the mesenchymal markers (*Vimentin, ZEB1, α‐SMA*, and *MMP1*) were increased. These results suggested that AHFS‐treated HEK underwent an epithelial‐mesenchymal transition process (Figure S7a, Supporting Information), which was essential for wound re‐epithelialization.^[^ ^46^ ^]^ The cell scratch experiment also showed that AHFS can promote HEK migration and wound healing (Figure S7b,c, Supporting Information). Likewise, the scratch experiment was also conducted to evaluate the effect of AHFS on HUVEC. Expectedly, AHFS exhibited the ability to facilitate HUVEC migration (Figure S7d,e, Supporting Information). These results demonstrated that, in addition to fibroblast‐DPC transition, AHFS can induce EMT in HEK and enhance the migration capability of HEK and HUVEC in vitro, which was of great significance for wound re‐epithelialization and healing. To investigate the wound repair potential of AHFS in vivo, a pilot full‐thickness excisional wound in the back skin was created, the wounds were photographed, and the wound area changes were recorded to vividly show the healing process. The vital applications of adhesive microspheres were investigated by quantifying the size of the wound healing area. As shown in **Figure** **8**a, we first created four groups (MS, TiT, AHFS, and PBS groups) of experiments to treat skin wounds. Then, at postoperative day (POD) 0, the TiT or PBS control was injected intradermally at the wound edge, and microspheres with or without TiT adhered to the wound (within 10 days). The healing wounds were harvested for further characterization at POD 6, 8, 10, 12, and 14. The accelerated wound healing of the AHFS group was observed as early as POD 6, the wound size of AHFS‐treated mice dropped significantly from 47.07 ± 3.4 mm^2^ to 8.15 ± 0.3 mm^2^, in which the average remaining area rate of the wound was 17.32% (Figure 8b–e). However, on POD 6, the wound sizes of PBS control, MS group, and TiT treatment groups were 17.68 ± 0.43, 17.97 ± 0.44, and 12.72 ± 0.35 mm^2^, respectively, with average wound remaining area of 37.78%, 37.89%, and 26.76%, respectively (Figure 8b–e). On POD 12, The wounds treated with AHFS were completely healed and covered with new epidermal tissue, but the wound sizes of PBS control, MS group, and TiT treatment groups were 5.45 ± 0.42, 3.48 ± 0.78, and 1.06 ± 0.16 mm^2^, respectively, in which 11.65%, 7.35%, and 2.23% of the wounds treated with PBS control, MS group, and TiT still remained unhealed (Figure 8b–e). For visualization, the dynamic healing process of each group at different times was traced in the schematic diagram in Figure 8c. To further test the in vivo function of AHFS for wound healing, H&E staining of the regenerated skin wounds on POD 14 was analyzed (Figure 8f). The results showed that wound re‐epithelialization was completed in all groups in all groups. In comparison with other groups, the wounds of the AHFS group displayed thicker but narrower granulation tissue, in which the average width of immature tissue was 0.45 mm, while that in the PBS control group, MS group, and TiT group and were 2.05, 1.75, and 0.95 mm, respectively (Figure 8g). It's worth noting that, on POD 14, a large number of immature hair follicles and reduced fibrosis was observed in TiT group and AHFS, while the PBS control group and MS group were not. These results suggested that AHFS could accelerate skin wound healing. Of note, AHFS‐treated wounds showed a faster healing rate than the TiT group and MS group, in which MS‐caused persistent release of TiT may mobilize wound‐resident cells for a long time.

### 2.7 Implant AHFS Yield Hair Follicle Regeneration In Situ Without Scarring

**Figure 9:**
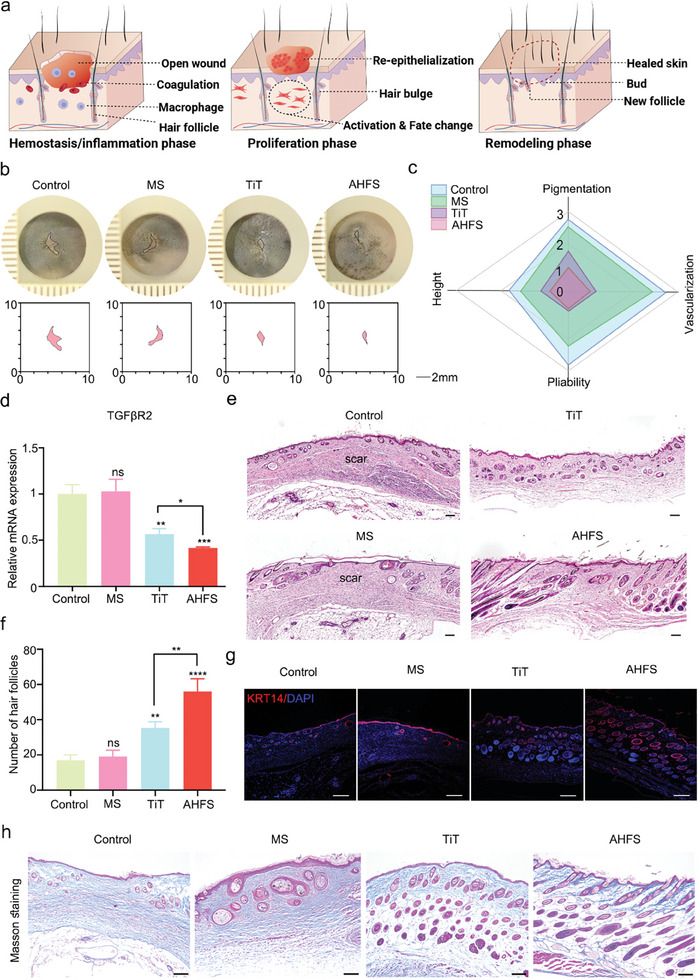
AHFS promote scarless wound healing and in situ hair follicle regeneration. a) Schematic illustration of the process of scar‐free repair, in vivo fibroblast‐DPC reprogramming and in situ hair follicle regeneration. b) Representative macroscopic illustration of scar on day 21 in control, MS, TiT and AHFS‐treated mice. c) Vancouver Scar Assessment Scale for evaluating scar on day 21 in control, MS, TiT and AHFS‐treated mice. d) qRT‐PCR analysis of transcriptional expression of profibrotic gene TGFβR2 in wounds of four groups at day 21. n = 3 per group. e) Hematoxylin and eosin (H&E) stained sections of wounds in four groups at day 21 showing scar tissue and hair follicle regeneration. Scale bar = 200 µm. f) Qualification of hair follicle numbers of wounds in four groups at day 21. n = 3 per group. g) Immunofluorescence images of KRT14 (red) in skin tissue within the wound area on day 21. h) Masson's trichrome‐stained skin tissue within the wound area on day 21. Scale bar = 100 µm. not significant, ^*^ *p* < 0.05, ^**^ *p* < 0.01, ^***^ *p* < 0.001, ^****^ *p* < 0.0001.

Given the results that AHFS can reprogram fibroblasts into DPC with the ability to regenerate hair follicles in vitro (**Figure** **9**a), a series of biological characteristics of regenerated skin tissues on POD 21 were analyzed, including scar evaluation, scar‐associated molecule level, the deposition of dermal collagen, DPC number and *de no* hair follicle neogenesis. On POD 21, the morphometric analysis showed that the scar area of the MS group was smaller than that in other groups (Figure 9b). The VSS scores (Scar Evaluation Scale) of the PBS control, MS group, TiT group, and AHFS group were 10, 10, 4, and 2, respectively (Figure 9c). The gene expression profiling related to scar formation of qRT‐PCR revealed significantly decreased mRNA levels of profibrotic factors (*TGFβR2*) in the AHFS group (Figure 9d). These results all demonstrated AHFS inhibited scar formation, and the mechanism may be associated with the reduced activation of fibroblast to myofibroblast. Then, the results of H&E staining suggested that there were more dense scar structures in the PBS control and MS group, while more *de no* hair follicle regeneration can be observed in the AHFS group and TiT group, and much more importantly, the AHFS group exhibited longer hair shaft than TiT group (Figure 9e,f). Cytokeratin 14 (KRT14) immunofluorescence further confirmed more dense and mature regenerated hair follicles after AHFS implantation for 21 days (Figure 9g). Besides, Masson's trichrome staining showed that AHFS treatment resulted in reduced collagen deposition and increased hair follicle regeneration (Figure 9h). In POD 28, the higher efficiency of hair regeneration was observed in AHFS treated group in the photos of healed wound (Figure S8a, Supporting Information). Furthermore, the skin tissue in POD 15 from all groups were analyzed by western blot, and the results showed that PI3K/AKT signaling pathway was also activated in AHFS group in vivo, which was consistent with that in vitro (Figure S8b,c, Supporting Information). Therefore, AHFS can effectively inhibit scarring, and promote the fibroblasts‐DPC transition and in situ hair follicle regeneration by PI3K/AKT signaling pathway.

## 3 Conclusion

The present study introduces the idea of functional skin repair by simultaneously interfering with the biological processes of scarring formation and HF regeneration. Herein, the seed microsphere AHFS was developed as the potential strategy to induce fibroblast‐to‐DPC reprogramming for inhibiting scar formation and promoting in situ HF regeneration. It has been proved that AHFS had wound‐adhesive characteristics, and it can circumvent the disadvantages of invasive therapy in early wound. In vitro studies demonstrated the AHFS could convert dermal fibroblasts to DPC by activating the PI3K/AKT signaling pathway. Besides, the slow‐released cocktail of AHFS also regulate fibroblast fate and enhanced wound‐resident cell ability in vivo, which can accelerate wound healing, and induce in situ HF regeneration with no scarring. This study comprehensively takes into account the process of scar formation and hair follicle regeneration, and used high‐throughput mechanism‐driven phenotype compound screening method, chemical reprogramming technology and biomaterial manufacturing, which may provide a new perspective in the biomaterial design for scarless wound healing and functional skin repair.

## 4 Experimental Section

All experiments related to animals and humans in this study were approved and carried out with approval from the Clinical Research Ethics Committee of the General Hospital of PLA (Beijing) and the Ethics Committee at the Fourth Medical Center of PLA General Hospital (approval No. 2019‐X‐15‐50). All the experimental details are reported in Supporting Information.

## Conflict of Interest

The authors declare no conflict of interest.

## Supporting information

Supporting Information

## Data Availability

The data that support the findings of this study are available in the supplementary material of this article.
