# Correction to “[Cocktail Cell‐Reprogrammed Hydrogel Microspheres Achieving Scarless Hair Follicle Regeneration]”

**DOI:** 10.1002/advs.202514108

**Published:** 2025-08-27

[Ji S, Li Y, Xiang L, Liu M, Xiong M, Cui W, Fu X, Sun X. Cocktail Cell‐Reprogrammed Hydrogel Microspheres Achieving Scarless Hair Follicle Regeneration. Adv Sci (Weinh). 2024, 11 (12), e2306305.]

https://doi.org/10.1002/advs.202306305

In Figure 4a,b, the image of LIVE/DEAD staining was incorrect and replaced. We conducted repeated experiments and proved the reliability of our results. The corrected data still revealed that AHFS seed microspheres had favorable biocompatibility with fibroblasts. The corrected figure is presented below.

Corrected Figure 4a,b


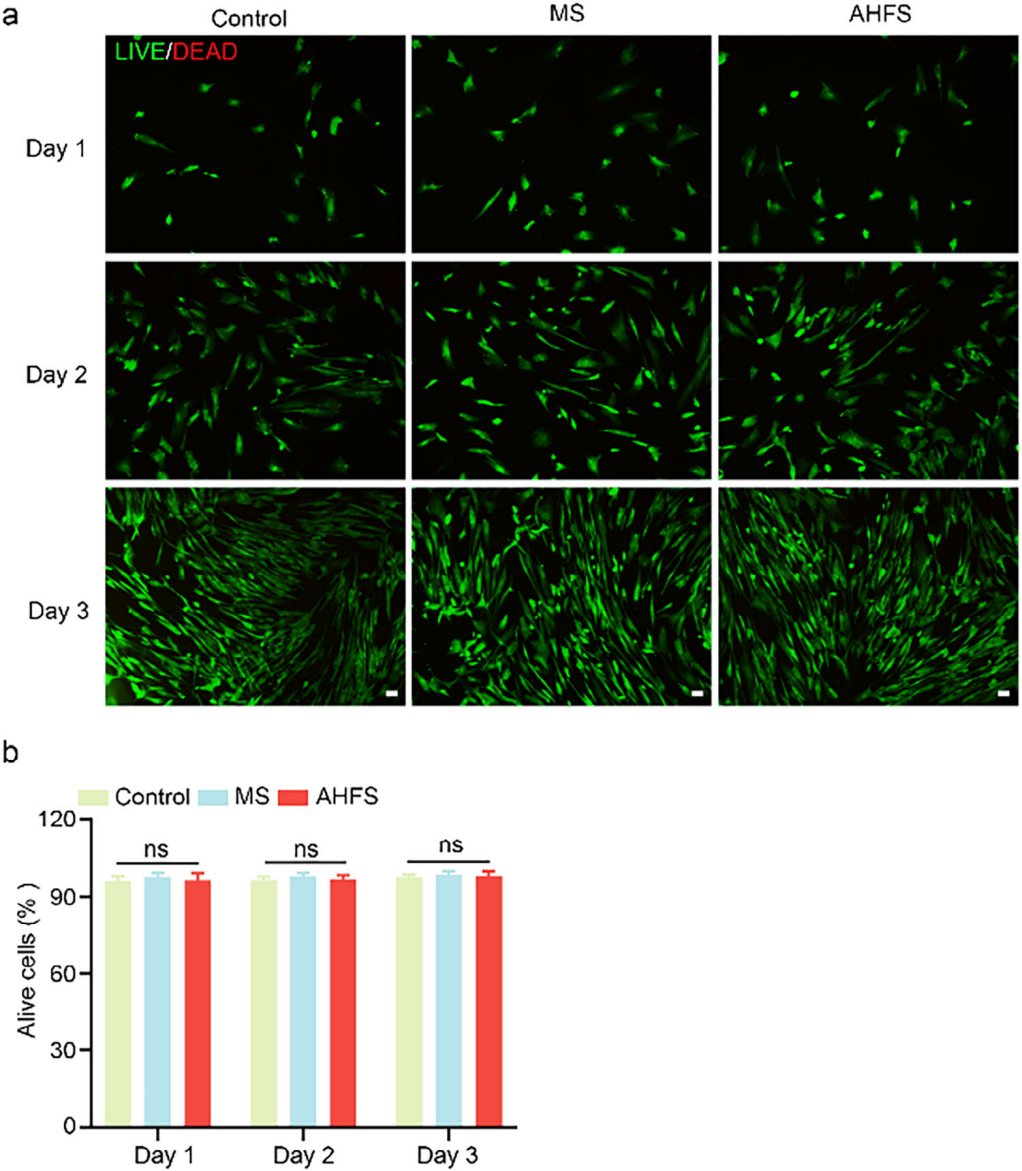


In Figure S1e (Supporting Information), the corrected result of GO enrichment in the fourth line is “positive regulation of cell population proliferation.”.

Corrected Figure S1e (Supporting Information)


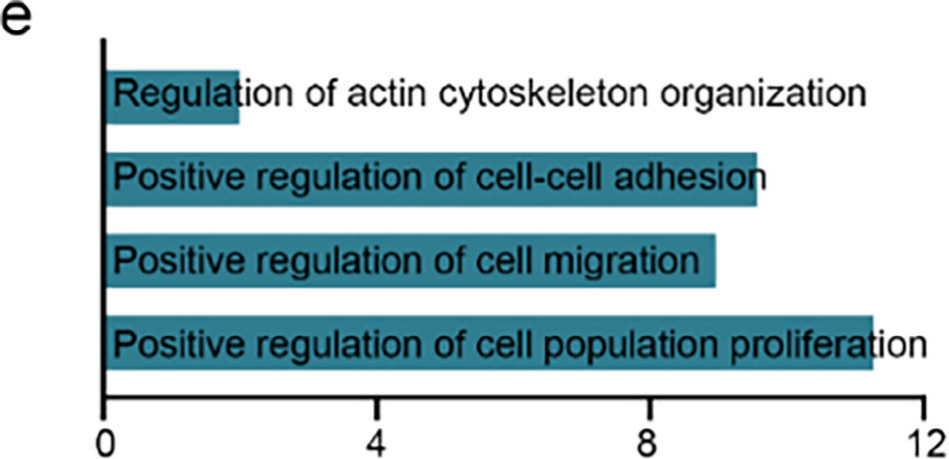


In Figure S4c,d (Supporting Information), the images of LIVE/DEAD staining were incorrect and replaced. We conducted repeated experiments and proved the reliability of our results. The corrected data still revealed that AHFS seed microspheres had favorable biocompatibility with HUVEC. The corrected figure is presented below.

Corrected Figure S4c,d (Supporting Information)


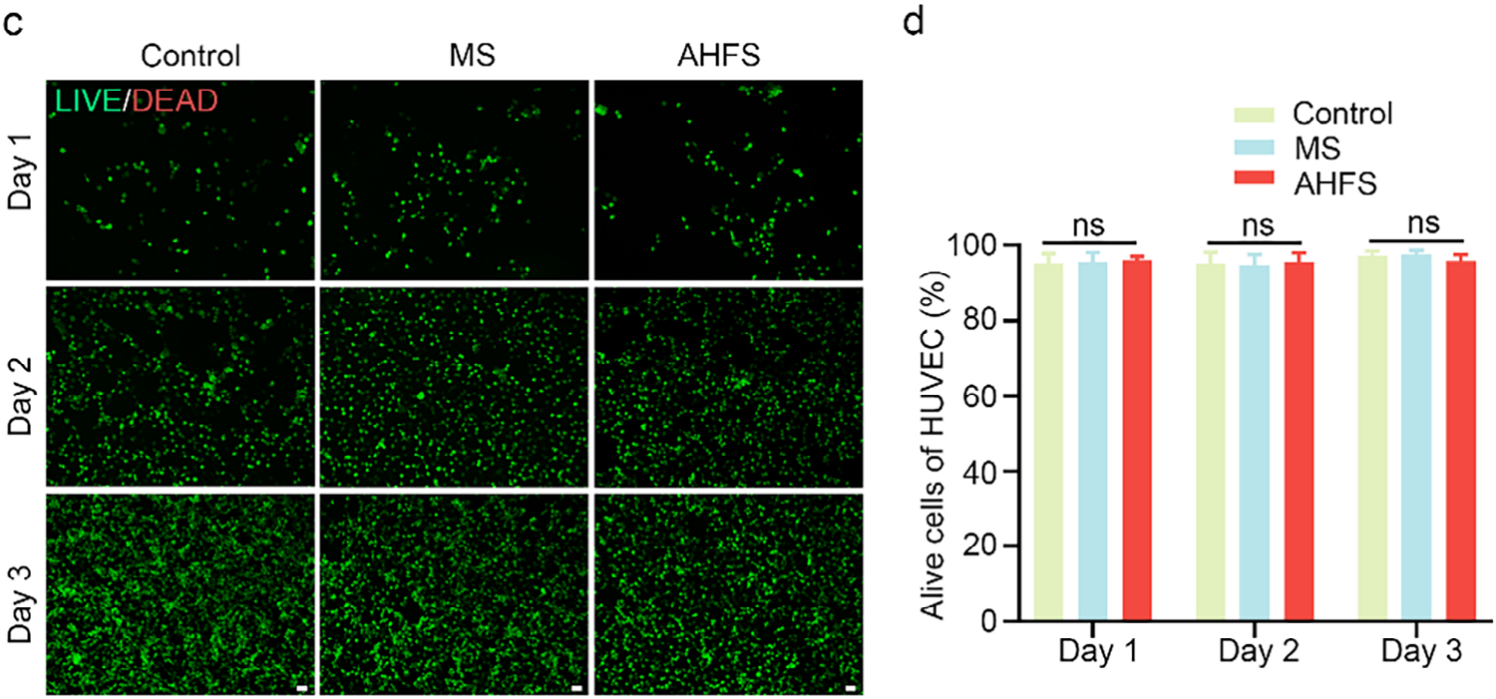


This correction does not affect the overall findings and conclusions of this paper. We apologize for these errors.

