# Supplementary material for: Cocktail Cell‐Reprogrammed Hydrogel Microspheres Achieving Scarless Hair Follicle Regeneration
Source: Adv Sci (Weinh). 2024 Jan 15;11(12):2306305. doi: 10.1002/advs.202306305 (PMC10966561; doi:10.1002/advs.202306305)
Supplement: Supplementary file 1 — Supporting Information [file ADVS-11-2306305-s001.pdf]

ADVANCED SCIENCE
Open Access

Supporting Information

for *Adv. Sci.*, DOI 10.1002/advs.202306305

Cocktail Cell-Reprogrammed Hydrogel Microspheres Achieving Scarless Hair Follicle Regeneration

Shuaifei Ji, Yingying Li, Lei Xiang, Mingyue Liu, Mingchen Xiong, Wenguo Cui, Xiaobing Fu* and Xiaoyan Sun**

Supporting Information

Cocktail cell-reprogrammed hydrogel microspheres achieving scarless hair follicle regeneration

Shuaifei Ji[*], Yingying Li[*], Lei Xiang [*], Mingyue Liu, Mingchen Xiong, Wenguo Cui[#], Xiaobing Fu[#], Xiaoyan Sun [#]

Dr. S. Ji[*], Dr. Y Li[*], Dr. M. Xiong, Pro. X. Fu[#], Pro. X. Sun[#]
Research Center for Tissue Repair and Regeneration affiliated to the Medical Innovation Research Department, PLA General Hospital and PLA Medical College; PLA Key Laboratory of Tissue Repair and Regenerative Medicine and Beijing Key Research Laboratory of Skin Injury, Repair and Regeneration; Research Unit of Trauma Care, Tissue Repair and Regeneration, Chinese Academy of Medical Sciences, 2019RU051, Beijing 100048, P. R. China
E-mail:fuxiaobing@vip.sina.com, yanzisun1979@sina.com.

Dr. L. Xiang[*], Dr. M. Liu, Pro. W. Cui[#].
Department of Orthopaedics, Shanghai Key Laboratory for Prevention and Treatment of Bone and Joint Diseases, Shanghai Institute of Traumatology and Orthopaedics, Ruijin Hospital, Shanghai Jiao Tong University School of Medicine, 197 Ruijin 2nd Road, Shanghai 200025, P. R. China.

E-mail: wgcui80@hotmail.com.

Experimental Section

Chemical cocktail screening

"Fibroblasts" and " Dermal papilla cells" as a search term in the GEO database (https://www.ncbi.nlm.nih.gov/geo/), the expression data of DPC and HDF was downloaded (GSE31324), which included 4 samples of “dermal papilla freshly-microdissected” (GSM776959-6962) and 4 samples of “fibroblasts cultured” (GSM776967-6970), and GPL571 was selected as the detection platform ([HG-U133A_2] Affymetrix Human Genome U133A 2.0 Array). GEO2R was used to identify the differentially expressed genes (DEG) of HDF and DPC. Firstly, background correction and normalization were performed on samples, and then DEG analysis was performed using the limma package of R language to determine the enriched genes. Log (Fold Change) >1 or < -1 and adjusted P<0.05 were considered as significantly expressed DEG. GO enrichment analysis and KEGG enrichment analysis of DEG were performed using STRING database (STRING: functional protein association networks (string-db.org))[1].

Connectivity Map [clue.io], a biological database of associations of genes, diseases, and drugs based on gene expression profiles, was as the platform for high-throughput mechanism-driven phenotype compound screening[2]. The principle of this database is to sequence thousands of drugs after treating different cells and record their gene expression profiles. Then, we can compare the list of differential genes with the reference data set of the database by sequencing the up-regulated and down-regulated DEG from tissue or cells. According to the enrichment of DEG in the reference gene expression profile, a correlation score (-100~100) was obtained. Positive numbers indicate that the up-regulated and down-regulated differentially expressed genes have similar expression profiles with reference genes, while negative numbers are the opposite. Ultimately, small molecule drugs that might play a role are sought based on the ratio of expression differences. Briefly, genome-wide chemical-induced gene expression is dependent on cMap for drug discovery or repurposing[3]. By uploading the DEG of HDF and DPC into the cMap database, a large number of drugs that may contribute to the conversion of HDF to DPC were

identified. Then, according to the reported mechanisms in cellular reprogramming, FDA approval status, and mRNA level of DPC-specific markers, optimized small molecular compounds with high reprogramming efficiency and clinical transformation prospect are gradually selected.

Preparation of the AHFS seed microspheres

Liposome carrying drugs were composed of lecithin, cholesterol, and fat-soluble drugs (Ti and T) that drive fibroblast fate toward DPC. The steps to synthesize liposomes are as follows. The mixture of 6.87 mg lecithin, 2.9 mg cholesterol, and moderate fat-soluble drugs was dissolved in 1 ml of chloroform and then evaporated for 20 min at 37℃ to form a uniform film. 1.5 ml deionized water was added and ultrasound with a probe (30% power) for 7min. Finally, the filters (0.45μm or 0.22μm) were used to remove free fat-soluble drugs. GelMA was prepared. Briefly, gelatin at a concentration of 10% (w/v) was completely melted in polyphosphate-buffered saline (PBS) at (60 °C, added into methacrylic acid (MA) solution, and mixed and reacted at 50°C for 4 h. An additional PBS diluent was used 5 times to stop the reaction, and then the compound was analyzed at 40°C for 1 week to filter impurities (14 kDa cut-off analysis tube). The GelMA aqueous solution was frozen and dried, resulting in a white, milky, porous structure foam. AHFS microspheres were prepared using an improved micro-liquid fluidity-focusing device. In simple terms, the aqueous phase (10 wt% GelMA and 2.5% liposome evenly mixed in PBS with 0.5% light stabilizer) and the oil phase (5 wt% Span80 in paraffin oil) were introduced into the micro-liquid device, and the water flow of the aqueous and oil phases was controlled using a syringe connected to a syringe pump. The resulting single-dispersive emulsion drops were optically cross-linked under UV light. The microspheres were placed into each microtubule. Then, 1 mL isopropyl alcohol was added, washed by oscillating and centrifuged at 4000 rpm to collect the microspheres. Subsequently, the microspheres were treated with 10% Poly allylamine hydrochloride (PAH) solution for 2h at 37° C under shaking at (")rpm. Then, the microspheres were washed to remove the excess dopamine.

Characterization of AHFS seed microspheres

The morphology and diameter of microspheres were determined by the bright-field microscope (LSM800, ZEISS, Germany). SEM (FEI Sirion 200, USA) was used to scan the surface morphology and microstructure of microspheres. Energy dispersive spectroscopy (EDS) and elemental mapping were utilized for the measurement of microsphere composition and element distribution. Microspheres were incubated with PBS containing hyaluronidase to assess its degradability, and the changes in microsphere morphology were monitored by microscopic observation at the indicated time. In the swelling test, 3mg microsphere is added to a 1.5mL tube and the weight of the microsphere and tube is measured before adding 1ml deionized water. Adjust the pH of the suspension to 7.4 and place the tube in a shaking incubator at 37°C and 80 rpm. At the specified time point, centrifuge the tube (3000 rpm, 3 min) and then remove the supernatant. Use filter paper to remove excess water before weighing. Repeat the whole process until you get a constant weight. For drug loading and release, all the chemicals used in this study were purchased from MCE, and the concentrations of the chemicals were measured via an ultraviolet spectrophotometer. Both Ti and T concentrations *in vitro* experiment were 5 μmol/L, while that were 50 μmol/L *in vivo* studies. Then, the microspheres were centrifuged and the unbound chemicals in the supernatant were collected to calculate the loading efficiency and loading capacity. The drug loading was calculated by ng/mg microsphere. The loaded microsphere was then incubated at 37℃ in 1ml PBS (pH = 7.4) containing 0.1% w/v BSA and stirred at 80 rpm. Chemicals released from the supernatant were measured at an indicated time point of 120 hours.

Cell Biocompatibility

Fibroblasts, epidermal keratinocytes, and vascular endothelial cells were all wound-resident cells that participate in wound healing. Considering the application of microspheres in the skin, the cell biocompatibility of microspheres with HDF, human epidermal keratinocyte (HEK), and human umbilical vein endothelial cells (HUVEC) were evaluated. All cells used in this study were obtained from the Cell Resource Center, Peking Union Medical College (http://cellresource.cn). HDF and HUVEC were cultured in DMEM/F12 containing 10% FBS and

1% penicillin & streptomycin (Solarbio, China). HEK were cultured in basic Epilife medium (Thermo Fisher Scientific) supplemented with 0.06 mm Ca^{2+}, 1% Epilife defined growth supplement (Thermo Fisher Scientific), and 1% penicillin & streptomycin (Solarbio, China). Briefly, co-culture of microspheres and HDF, HEK, and HUVEC for 3 days, and cellular viability was evaluated by live/Dead staining assay. On days 1, 2, and 3, the cells co-cultured with microspheres were treated with 250 µL Calcein AM/PI detection working solution (Solarbio, China) for 30 min and subsequently observed under a fluorescent microscope. Because HDF were the targeted cells of microspheres, cytoskeleton staining, and the cellular proliferation on days 1, 2, and 3, were further measured using TRITC Phalloidin (CA1610, Solarbio, China) and Cell Count Kit-8 (CCK-8, Solarbio, China), respectively. The HDF and HEK used in this study were obtained from clinical tissue, and HUVEC was purchased from the National Infrastructure of Cell Line Resource. The present studies were approved by the Clinical Research Ethics Committee of the General Hospital of PLA (Beijing, China), and written informed consent was obtained from all individuals before samples were obtained.

Quantitative real-time PCR

According to the manufacturer's advice, using TRIzol reagent (Invitrogen, USA) to extract total RNA from cells or tissues. Reversing transcription of total RNA (500 ng) with a PrimeScript RT reagent kit (TaKaRa, Japan) to generate cDNA. In quantitative real-time polymeric chain enzyme reaction (qRT-PCR), targeted genes were analyzed quantitatively by SYBR Green Supermix (Bio-Rad, USA). The details of primer information were listed in Table S2.

ALP staining

For detection of ALP activity to evaluate the characteristics of AHFS-treated cells, the HDF and microsphere-treated cells (1x 10^6) were fixed with 4% paraformaldehyde for 10 min and then incubated with ALP staining solution (BCIP/NBT alkaline phosphatase color development kit, C3206, Beyotime) for 30 min at room temperature away from light. The color reaction can be terminated by removing the ALP staining solution and washing 1-2 times with distilled water.

Immunofluorescence

Cells or tissues were fixed in 4% paraformaldehyde at room temperature for 30 min, then were permeabilized in 0.2% Triton ×100 in PBS for 10 min, and finally, were incubated in 1x PBS containing 10 % normal goat serum to block non-specific protein-protein interaction followed by primary antibodies at 4 °C overnight and secondary antibodies at room temperature for 2 h. The primary antibodies used in this study were mouse anti-α-SMA (ab7817, 1:200, Abcam) and rabbit anti-CK14 (ab119695, 1:200, Abcam). The following secondary antibodies were used: goat anti-mouse IgG H&L (Alexa Fluor 488) (ab150113, Abcam, 1:200), goat anti-rabbit IgG H&L (Alexa Fluor 594) (ab150080, 1:200, Abcam). Visualization was performed on a Leica fluorescence microscope.

Cell cycle analysis

Cells were fixed in 70% precooled ethanol at 4 °C overnight. After washing with PBS, cells were re-suspended with 100μl RNase A solution in 37°C water baths for 30min, followed by 400μl PI staining solution at 4 °C for 30 min. Finally, the treated cell suspension was detected and the red fluorescence at 488nm was recorded.

Western blotting

Using RIPA buffer (Sigma-Aldrich) to obtain total protein from cells or tissue. Then, the protein was separated by SDS Polyacrylamide gel electrophoresis (SDS-PVGE) and transferred to PVDF membranes (GE Healthcare). Protein bands were incubated with the following primary antibodies overnight at 4 °C: mouse anti-α-SMA (ab7817, 1:1000, Abcam), rabbit anti-p-AKT (4060S, 1:1000, CST), rabbit anti- AKT (4691S, 1:1000, CST), rabbit anti-β-CATENIN (ab32572, 1:1000, Abcam) and mouse anti-GAPDH (60004-1-Ig, 1:50000, proteintech). The blots were then washed and incubated with horseradish peroxidase-conjugated secondary antibodies (sc-2005, sc-2004, Santa Cruz, 1:1000). Protein bands were detected by enhanced chemiluminescence (ECL) detection reagent.

Full-thickness wound model

For plot full-thickness excisional wound-healing assays, 8-week-old female C57BL/6 mice (n=20) were used for wound healing assay. Briefly, 8 mm diameter

full-thickness excisional wounds were created on the back of mice, and treatments of the mice were randomly divided into four groups: MS, TiT, AHFS, and PBS groups. The wound mice (n=5/group) were given a subcutaneous injection of 100 μL PBS and TiT solution (Ti=50μmol/L, T=50μmol/L), and a wound surface adhesion of 500 μL MS (80/mL) and AHFS (80/mL, Ti=50μmol/L, T=50μmol/L) within 10 days. The PBS and TiT solution (100 μL) were injected subcutaneously around the wounds every 5 days (until 15 days). The pictures of the wounds were taken on days 0, 6, 8, 10, and 12 by a digital camera. Mice were monitored daily and sacrificed at indicated days. Wound remaining area (%) = (actual wound area/initial wound area) × 100%.

Statistical analysis

All data shown in the graphs are presented as mean ± S.D.. The data follows a normal distribution and the variances were homogeneous. Statistical analysis was performed with GraphPad Prism 8.0 software. Comparisons between 2 groups were analyzed by two-sided test, and one-way ANOVA was used for more than two groups. The value of $p<0.05$ was considered statistically significant. ns represented no significant difference; *$P < 0.05$;**$P < 0.01$;***$P < 0.001$;****$P < 0.0001$.

References

[1] D. Szklarczyk, A. L. Gable, K. C. Nastou, D. Lyon, R. Kirsch, S. Pyysalo, N. T. Doncheva, M. Legeay, T. Fang, P. Bork, L. J. Jensen, C. von Mering, *Nucleic Acids Res* **2021**, 49, D605.

[2] A. Subramanian, R. Narayan, S. M. Corsello, D. D. Peck, T. E. Natoli, X. Lu, J. Gould, J. F. Davis, A. A. Tubelli, J. K. Asiedu, D. L. Lahr, J. E. Hirschman, Z. Liu, M. Donahue, B. Julian, M. Khan, D. Wadden, I. C. Smith, D. Lam, A. Liberzon, C. Toder, M. Bagul, M. Orzechowski, O. M. Enache, F. Piccioni, S. A. Johnson, N. J. Lyons, A. H. Berger, A. F. Shamji, A. N. Brooks, A. Vrcic, C. Flynn, J. Rosains, D. Y. Takeda, R. Hu, D. Davison, J. Lamb, K. Ardlie, L. Hogstrom, P. Greenside, N. S. Gray, P. A. Clemons, S. Silver, X. Wu, W. N. Zhao, W. Read-Button, X. Wu, S. J. Haggarty, L. V. Ronco, J. S. Boehm, S. L. Schreiber, J. G. Doench, J. A. Bittker, D. E. Root, B. Wong, T. R. Golub, *Cell* **2017**, 171, 1437.

[3] A. Musa, L. S. Ghoraie, S. D. Zhang, G. Glazko, O. Yli-Harja, M. Dehmer, B. Haibe-Kains, F. Emmert-Streib, *Brief Bioinform* **2018**, 19, 506.

Supporting figures

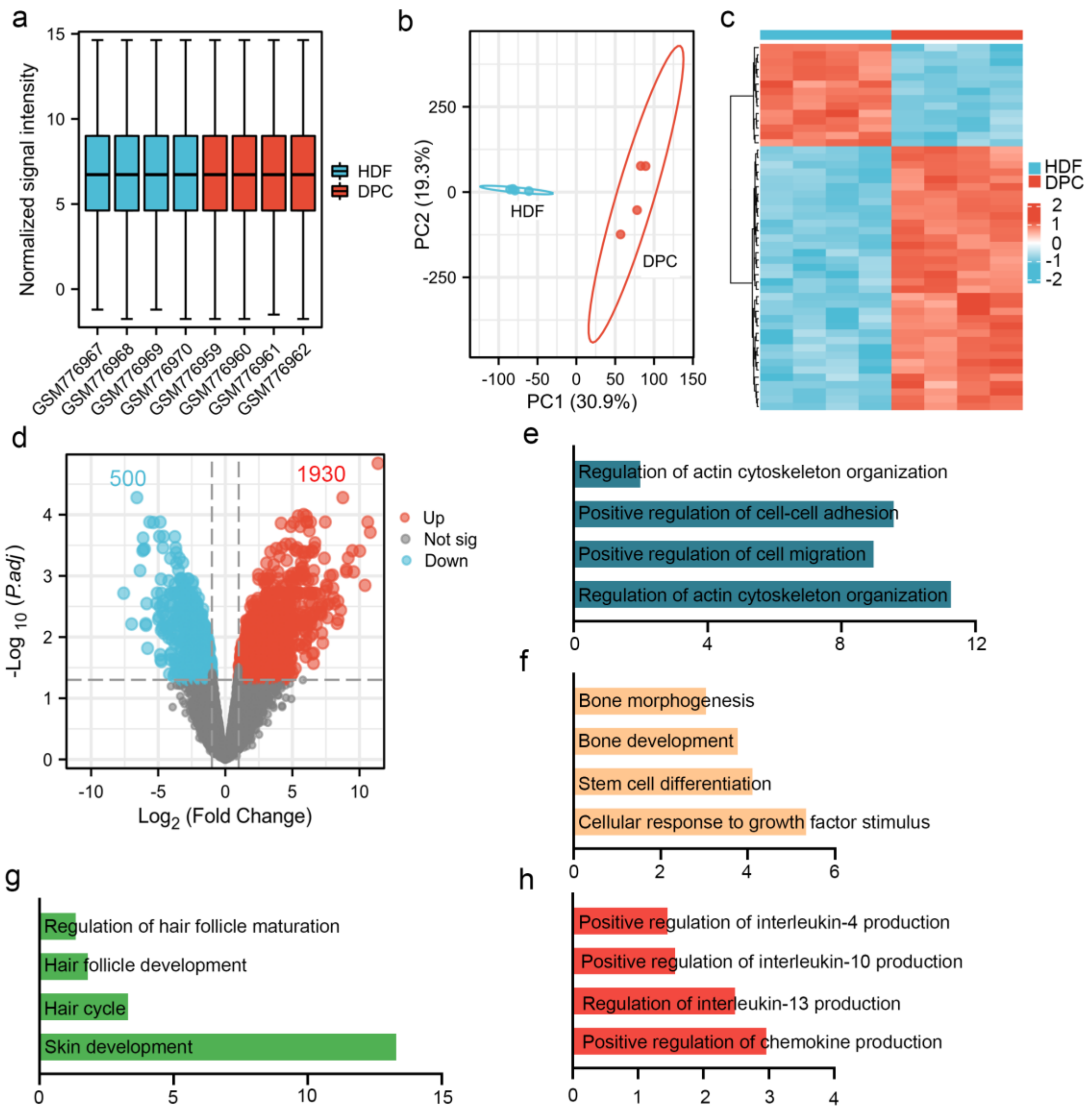

Figure S1. Transcriptomic differences based on Transcriptomic s between primary DPC and HDF**.** a) Normalized signal intensity of HDF and DPC samples, showing the good uniformity. b) Principal Components Analysis (PCA) of HDF and DPC samples. c) Heatmap analysis between HDF and primary DPC. d) Volcano plot showing the differentially expressed genes (DEG) of primary DPC compared to HDF. Not sig, not significant. Up-regulated DEG enriched in DPC function and feature: e) cell activity. f) Osteogenic differentiation and stem cell property. g) Hair follicle and skin development. h) Interleukin and chemokine production.

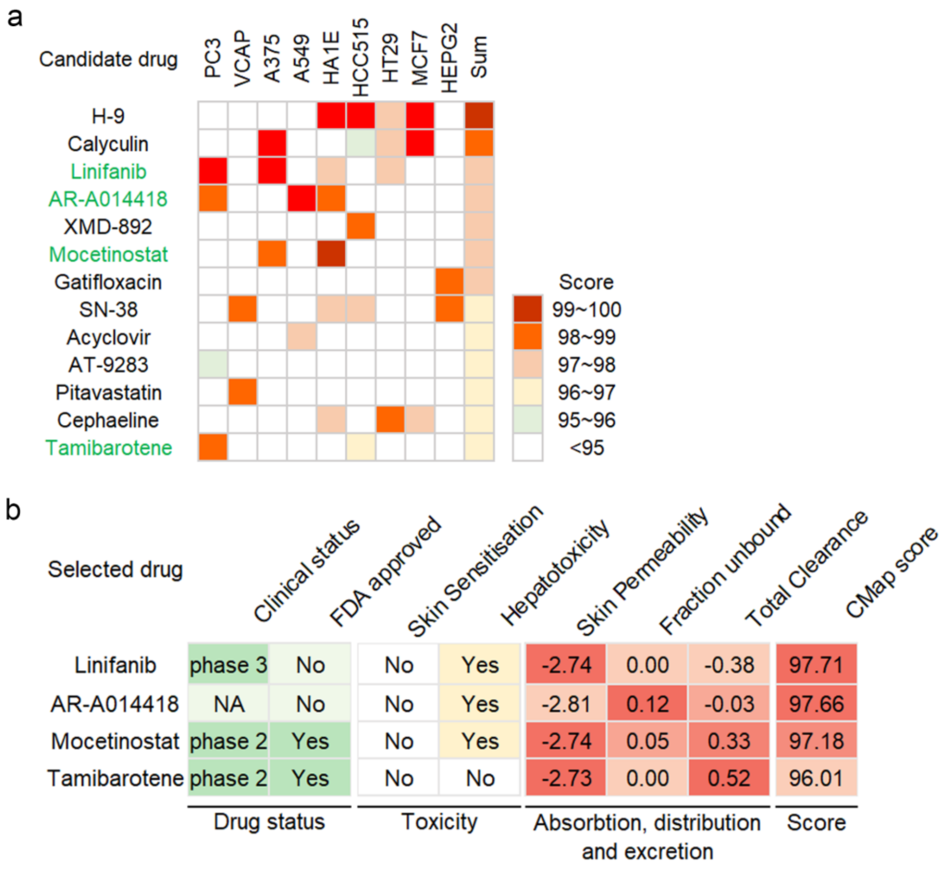

Figure S2. The information of predicted drug candidates that induce fibroblast-DPC transition. a) The cMap score of drug candidates**.** b) Clinical status and biochemical characteristics of drug candidates.

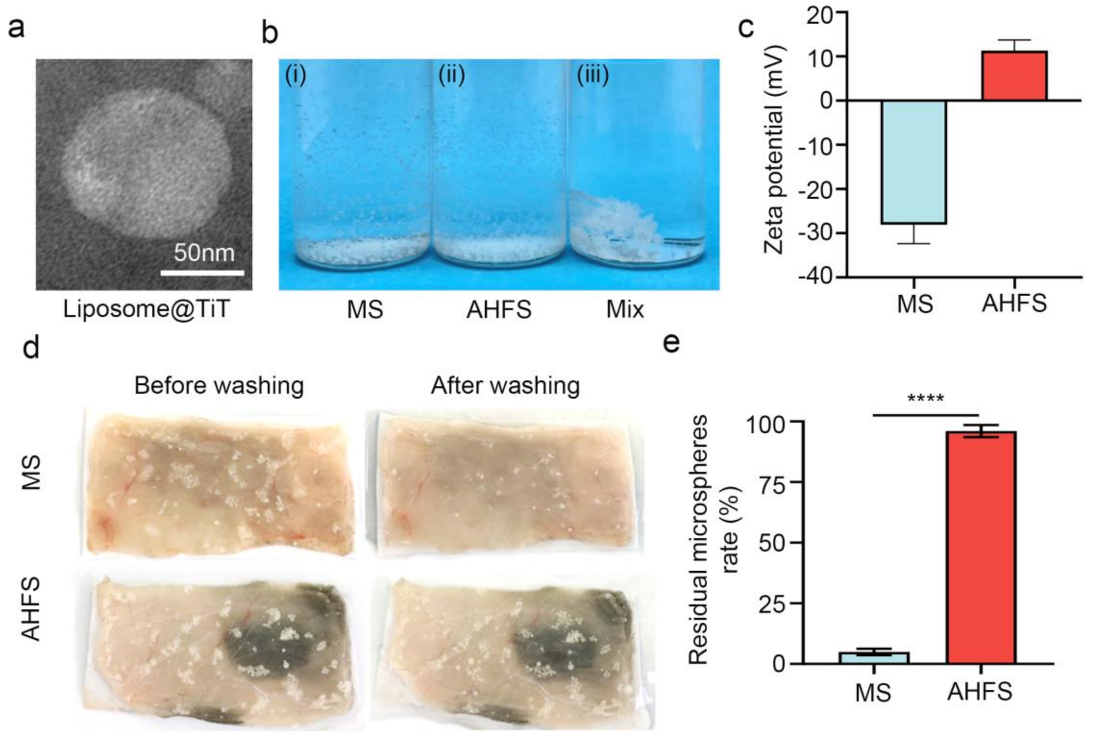

Figure S3. The property of AHFS microspheres. a) The micromorphology of drug-loaded liposomes; b) Macroscopic and local images of microspheres, AHFS, and a mixture of both; c) the zeta potential of the microspheres without polyamide modification and AHFS; d) Images of microspheres adhesion on the skin surface of mice before and after rinsing with running water; e) The number of microspheres remaining on the skin surface after rinsing. As the control, MS in this experiment was non-adhesive. Data are expressed as mean ± S.D.; n = 3. ***, $p < 0.001$.

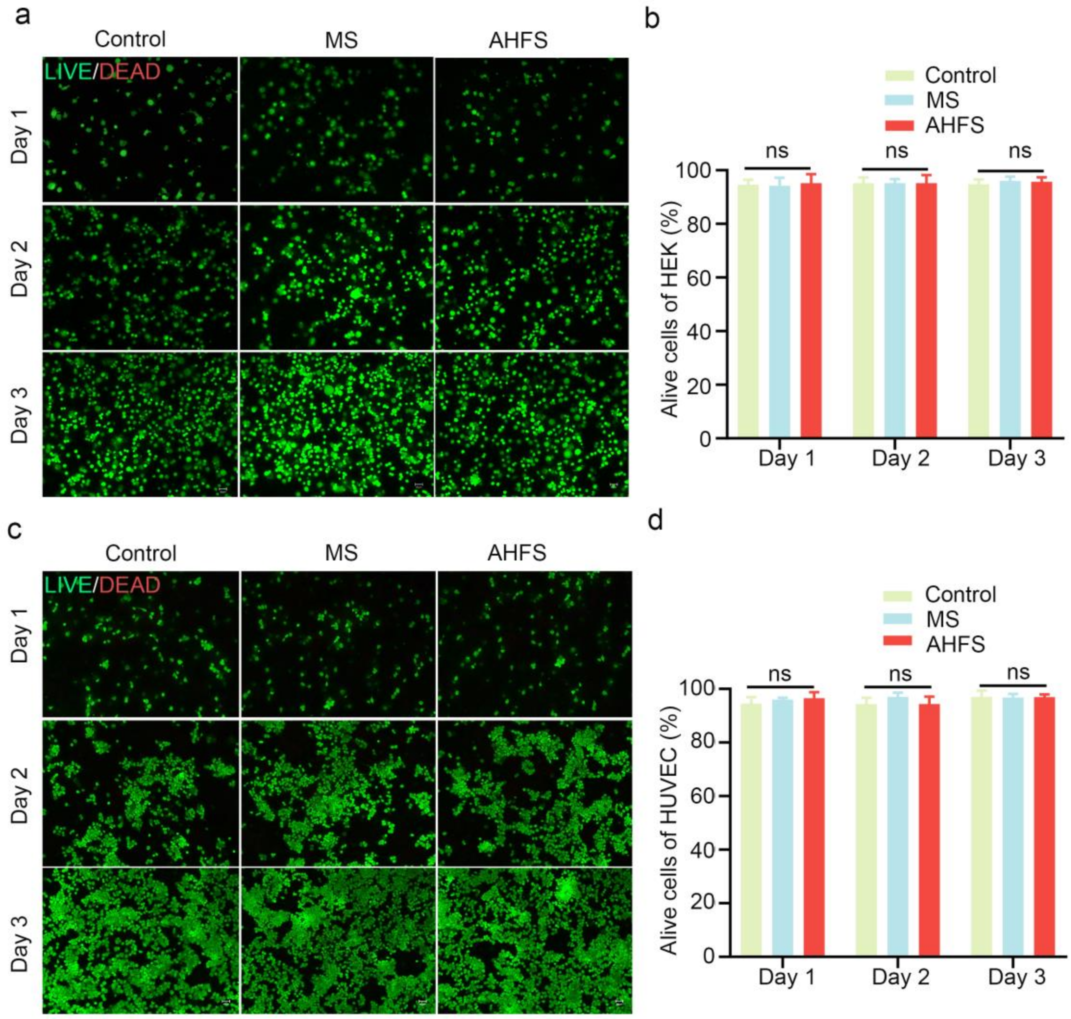

Figure S4. The biocompatibility of AHFS microspheres. a) Images of HEK live/dead cell staining on day 1, day2 and day 3, among control, MS microsphere and AHFS microsphere group. Scale bar = 50 μm. b) Quantitative analysis for the percentage of alive HEK cells in control, MS microsphere, and AHFS microsphere group. Results are expressed as mean ± S.D.; ns, not significant. c) Images of HUVEC live/dead cell staining on day 1, day2 and day 3, among control, MS microsphere and AHFS microsphere group. Scale bar = 50 μm. d) Quantitative analysis for the percentage of alive HUVEC cells in control, MS microsphere, and AHFS microsphere group. Results are expressed as mean ± S.D.; ns, not significant.

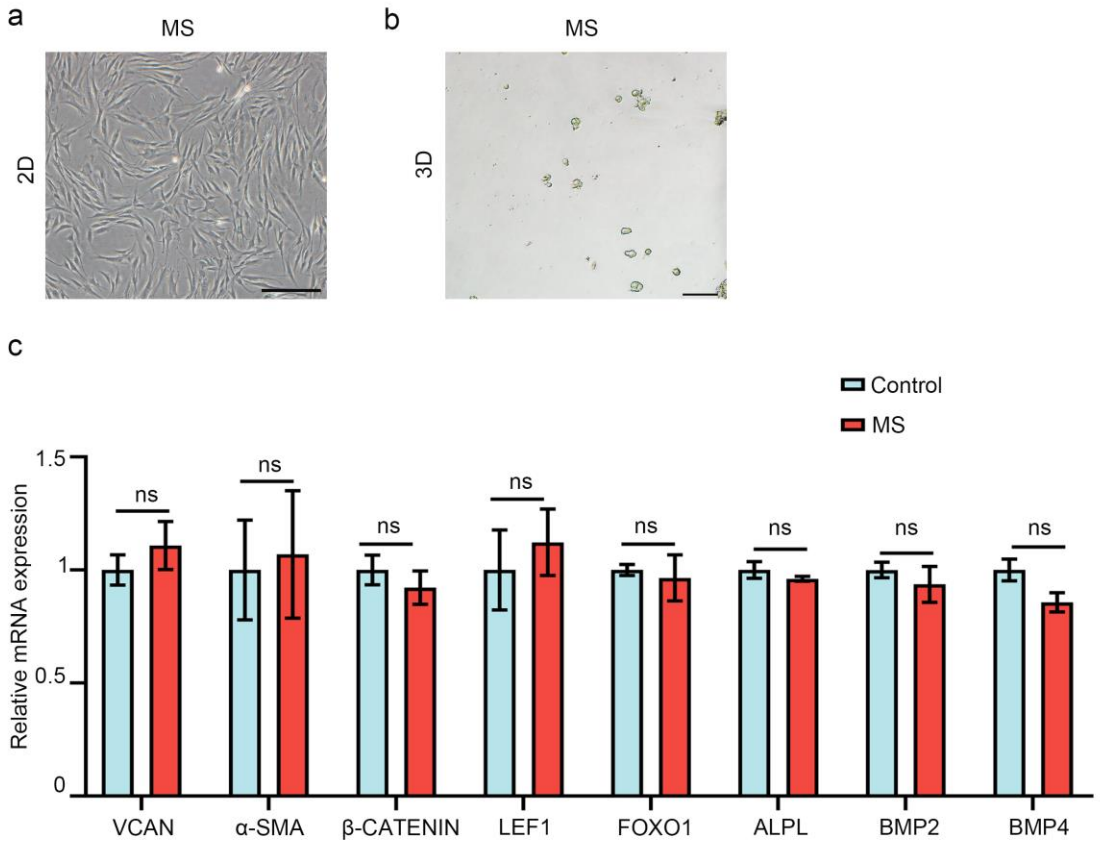

Figure S5. The effect of MS microspheres on HDF. a) Phase contrast images showing the morphological characteristics of HDF and MS microsphere-treated DPC in 2D culture. Scale bar = 200 µm. b) Multicellular sphere formation assay of HDF and MS microsphere-treated DPC in 3D culture. Scale bar = 100 µm. c) qRT-PCR analysis of transcriptional expression of DPC-associated maker α-SMA, CXCR4, BMP2, BMP4, ALPL, LEF1, β-CATENIN and FOXO1 in HDF and MS microsphere-treated DPC after 5 days of induction. All data in qRT-PCR analysis are expressed as mean ± S.D.; n = 3. ns, not significant.

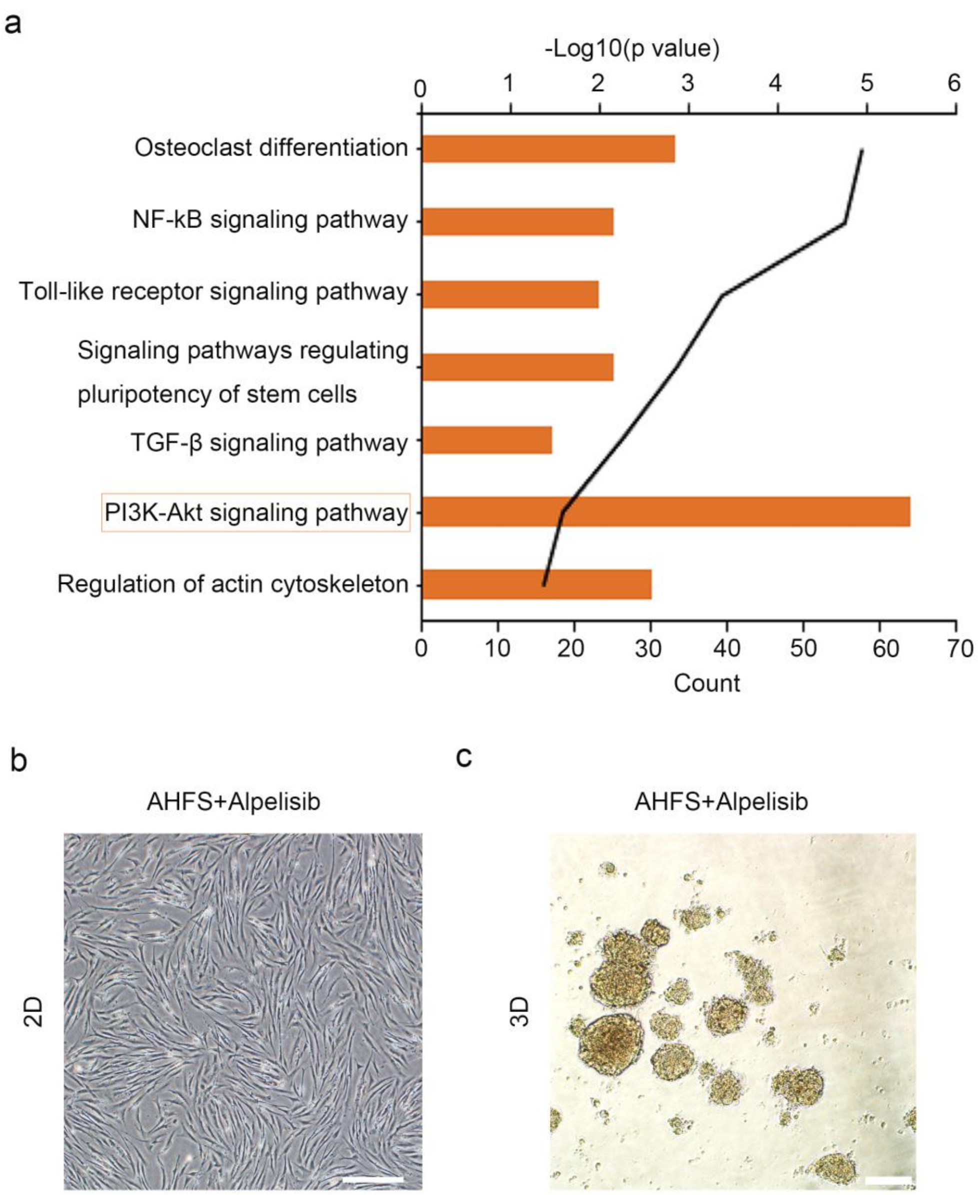

Figure S6. PI3K inhibitor blocked the AHFS microspheres-induced fibroblast-DPC transition. a) KEGG analysis of upregulated DEG between primary DPC and HDF, showing PI3K/AKT signaling pathway was significantly enriched. b) Phase contrast images showing the morphological characteristics of HDF with or without AHFS microsphere+Alpelisib treatment in 2D culture. Scale bar = 200 µm. c) Multicellular sphere formation assay of HDF with or without AHFS microsphere+Alpelisib treatment in 3D culture. Scale bar = 100 µm

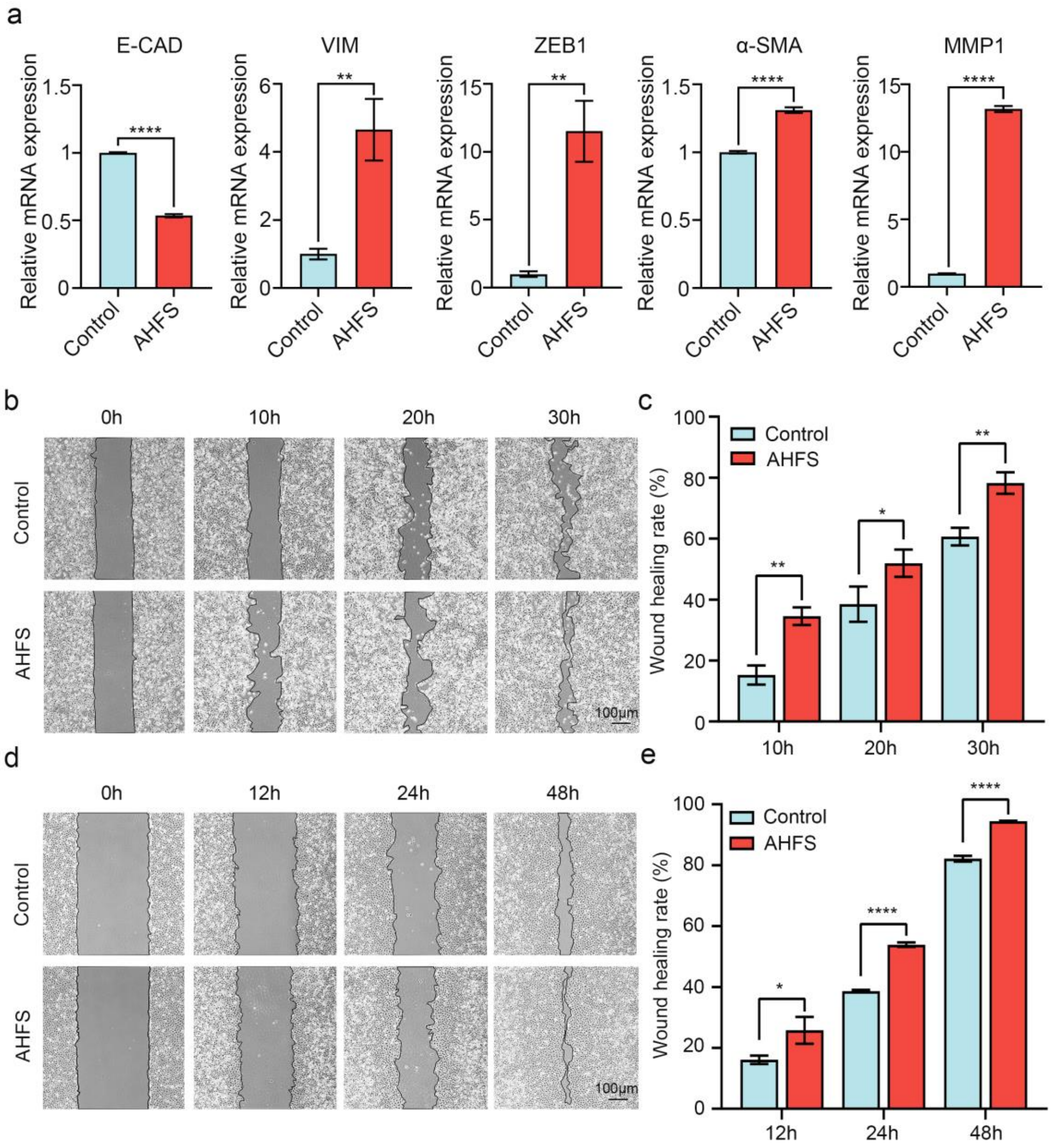

Figure S7. The effect of AHFS microspheres on HEK and HUVEC. a) qRT-PCR analysis of transcriptional expression of EMT-associated maker E-CAD, VIM, ZEB1, α-SMA, and MMP1 in HEK with or without AHFS microsphere treatment. All data in qRT-PCR analysis are expressed as mean ± S.D.; n = 3. **, $p < 0.01$, ****, $p < 0.0001$. b) Representative images of HEK scratch assay with or without AHFS microsphere treatment. black dotted lines mark the scratch edges. Scale bar = 100 μm. c) Histogram showing percentage of the denuded area covered by migrated HEK to the original scratch area. d) Representative images of HUVEC scratch assay with or without AHFS microsphere treatment. black dotted lines mark the scratch edges.

Scale bar = 100 µm. e) Histogram showing percentage of the denuded area covered by migrated HUVEC to the original scratch area. black dotted lines mark the scratch edges. All data from three independent experiments were presented as mean ± SD.; n = 3. *, $p < 0.05$, **, $p < 0.01$, ****, $p < 0.0001$.

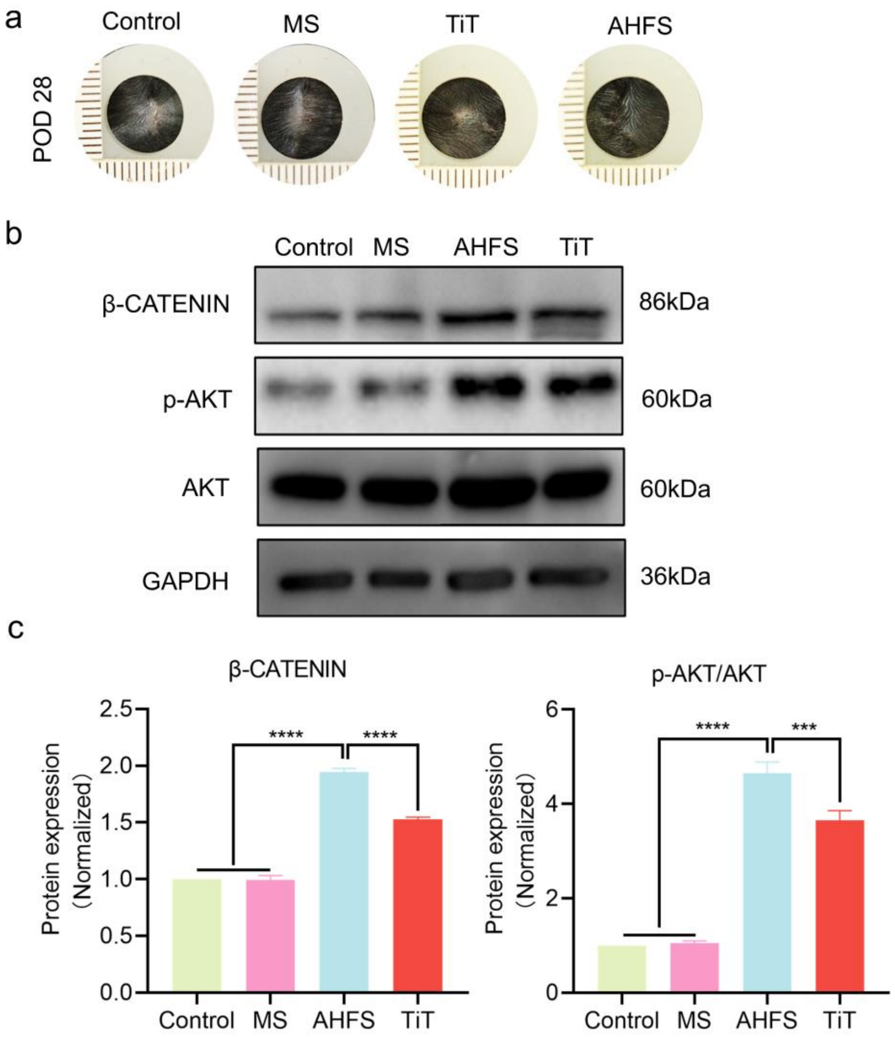

Figure S8. Hair follicle regeneration and *in vivo* mechanism verification. a) The efficiency of hair regeneration in healed wound (POD 28); b) Western blot analysis of wound tissue in phosphorylation AKT, AKT and DPC marker β-CATENIN in control group, MS group, AHFS group and TiT group; c) Qualification of β-CATENIN, and p-AKT/AKT immunoblots was performed using Image J software. GAPDH was used as internal loading control. ***, $p < 0.001$, ****, $p < 0.0001$.

Table S1. Chemicals from cMap database based on the transcriptomic analysis of HDF and primary DPC

Name	ID	Description	Target	Structure
H-9	BRD-K70577657	PKA inhibitor	PRKACA	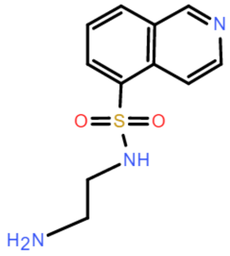
Calyculin	BRD-A47513740	Protein phosphatase inhibitor	PPP1CA, PPP1CC, PPP2CA	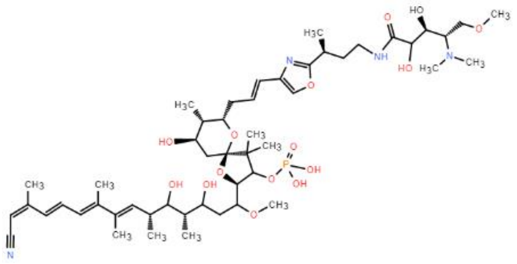
Linifanib	BRD-K99749624	PDGFR receptor inhibitor	CSF1R, KDR, PDGFRB, FLT1, FLT3, FLT4, CSF1, KIT, PDGFRA, RET, TEK	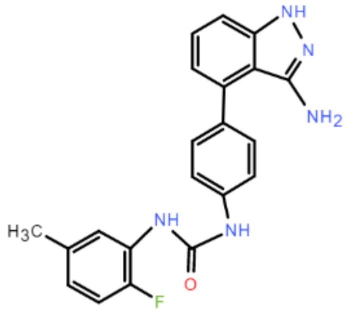

AR-A014418	BRD-K67860401	Glycogen synthase kinase inhibitor	GSK3β	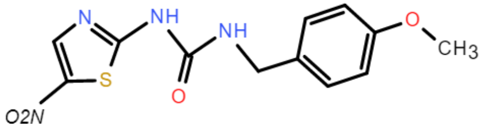
XMD-892	BRD-K50387473	MAP kinase inhibitor	MAPK7, DCLK2, LRRK2, PLK4, TNK1	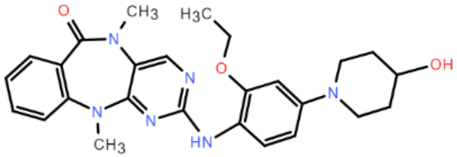
Mocetinostat	BRD-K16485616	HDAC inhibitor	HDAC1, HDAC2, HDAC3, HDAC11	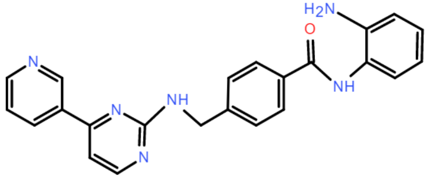
Gatifloxacin	BRD-A74980173	Bacterial DNA gyrase inhibitor	TOP2	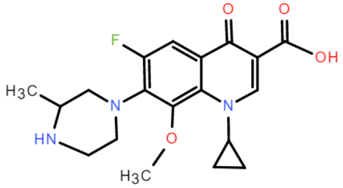
SN-38	BRD-A36630025	Topoisomerase inhibitor	TOP1	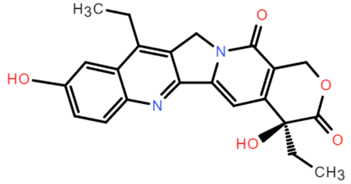

Acyclovir	BRD-K32318651	DNA polymerase inhibitor	PNP	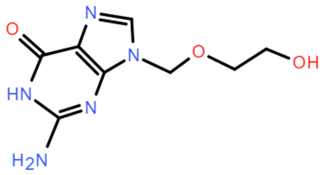
AT-9283	BRD-K24576554	JAK inhibitor	AURKA, AURKB, ABL1, BCR, FLT3, JAK2, JAK3, RPS6KA6, STK17A	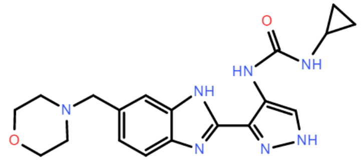
Pitavastatin	BRD-K30097969	HMGCR inhibitor	HMGCR, APOA1, CYP2C8	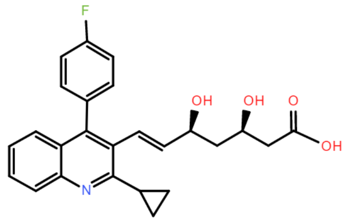
Cephaeline	BRD-K80348542	Protein synthesis inhibitor	RPS2	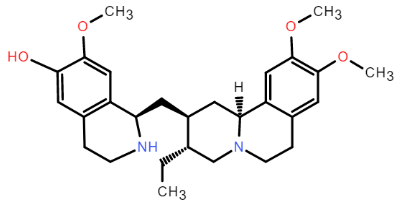
Tamibarotene	BRD-K36627727	Retinoid receptor agonist	RARA, RARB	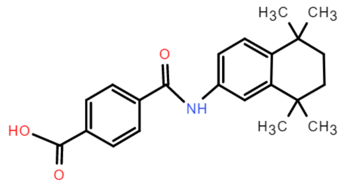

Table S2. Primer sequences used in the study

Genes	Forward primer	Reverse primer
β-ACTIN	CATGTACGTTGCTATCCAGGC	CTCCTTAATGTCACGCACGAT
E-CAD	CGAGAGCTACACGTTCACGG	GGGTGTCGAGGGAAAAATAGG
VIM	GACGCCATCAACACCGAGTT	CTTTGTCGTTGGTTAGCTGGT
ZEB1	GATGATGAATGCGAGTCAGATGC	ACAGCAGTGTCTTGTTGTTGT
MMP1	AAAATTACACGCCAGATTTGCC	GGTGTGACATTACTCCAGAGTTG
VCAN	GTAACCCATGCGCTACATAAAGT	GGCAAAGTAGGCATCGTTGAAA
CXCR4	ACTACACCGAGGAAATGGGCT	CCCACAATGCCAGTTAAGAAGA
BMP2	ACCCGCTGTCTTCTAGCGT	TTTCAGGCCGAACATGCTGAG
BMP4	ATGATTCCTGGTAACCGAATGC	CCCCGTCTCAGGTATCAAACT
ALPL	ACCACCACGAGAGTGAACCA	CGTTGTCTGAGTACCAGTCCC
LEF1	AGAACACCCCGATGACGGA	GGCATCATTATGTACCCGGAAT
β-CATENIN	AAAGCGGCTGTTAGTCACTGG	CGAGTCATTGCATACTGTCCAT
FOXO1	TCGTCATAATCTGTCCCTACACA	CGGCTTCGGCTCTTAGCAAA
WNT7A	CTGTGGCTGCGACAAAGAGAA	GCCGTGGCACTTACATTCC
WNT5A	ATTCTTGGTGGTCGCTAGGTA	CGCCTTCTCCGATGTACTGC
WNT10A	GGTCAGCACCCAATGACATTC	TGGATGGCGATCTGGATGC
KRT15	GACGGAGATCACAGACCTGAG	CTCCAGCCGTGTCTTTATGTC
LGR6	AGCCCTGTGAGTACCTCTTTG	CCACAGGAAATGCCAGTCAA
TGFβR2	GACTGTCCACTTGCGACAAC	GGCAAACCGTCTCCAGAGTAA

E-CAD, E-cadherin; VIM, Vimentin; α-SMA, Alpha-skeletal muscle actin; ZEB1, Zinc finger E-box binding homeobox 1; MMP1, Matrix metallopeptidase 1; VCAN, Versican; CXCR4, C-X-C motif chemokine receptor 4; BMP2, Bone morphogenetic protein 2; BMP4, Bone morphogenetic protein 4; ALPL, Alkaline phosphatase; LEF1, Lymphoid enhancer binding factor 1; FOXO1, Forkhead box O1; WNT7A, Wnt family member 7A; WNT5A, Wnt family member 5A; WNT10A, Wnt family member 10A; KRT15, Keratin 15; LGR6, Leucine rich repeat containing G protein-coupled receptor 6; TGFβR2, Transforming growth factor beta receptor 2.
